# MIEF1/2 function as adaptors to recruit Drp1 to mitochondria and regulate the association of Drp1 with Mff

**DOI:** 10.1038/s41598-017-00853-x

**Published:** 2017-04-13

**Authors:** Rong Yu, Tong Liu, Shao-Bo Jin, Chenfei Ning, Urban Lendahl, Monica Nistér, Jian Zhao

**Affiliations:** 1 Department of Oncology-Pathology, Karolinska Institutet, CCK R8:05, Karolinska University Hospital Solna, SE-171 76 Stockholm, Sweden; 2 Department of Cell and Molecular Biology, Karolinska Institutet, SE-171 77 Stockholm, Sweden

## Abstract

Mitochondrial dynamics is a fundamental cellular process and recruitment of Drp1 to mitochondria is an essential step in mitochondrial fission. Mff and MIEF1/2 (MiD51/49) serve as key receptors for recruitment of Drp1 to mitochondria in mammals. However, if and how these receptors work together in mitochondrial fission is poorly understood. Here we show that MIEFs interact with both Drp1 and Mff on the mitochondrial surface and serve as adaptors linking Drp1 and Mff together in a trimeric Drp1-MIEF-Mff complex. Thus, MIEFs can regulate the interaction between Drp1 and Mff, and also Mff-induced Drp1 accumulation on mitochondria. It is shown that loss of endogenous MIEFs severely impairs these processes. Additionally, in cells depleted of endogenous MIEF1/2, high levels of exogenous MIEFs sequester Drp1 on the mitochondrial surface, resulting in mitochondrial elongation, whereas low-to-moderate levels of MIEFs promote mitochondrial fission, leading to mitochondrial fragmentation. In sum, the data suggest that MIEFs and Mff work coordinately in Drp1-mediated mitochondrial fission and that the level of MIEF1/2 relative to Mff sets the balance between mitochondrial fission and fusion.

## Introduction

Cells need to regulate the morphology of mitochondria in response to various physiological challenges and the dynamin**-**related GTPase Drp1 has emerged as a central regulator in mitochondrial fission. Drp1 is primarily distributed in the cytoplasm, but shuttles between the cytoplasm and mitochondria^1, 2^. Drp1 recruitment from the cytoplasm to the mitochondrial outer membrane (MOM) is an essential step in mitochondrial fission^3–5^. At the MOM, Drp1 is assembled into helical structures that wrap around the mitochondria to induce mitochondrial fission via its GTPase activity^1, 5, 6^. Several proteins located at the MOM, including Fis1, Mff and MIEFs (MIEF1 and MIEF2, also known as MiD51/MiD49) have been identified as receptors for the recruitment of Drp1 to mitochondria in mammals. While Fis1 was the first proposed Drp1 receptor at the MOM^7, 8^, several recent studies suggest that Fis1 plays only a minor role in Drp1 recruitment^9–11^. Mff and MIEFs have been identified as alternative receptors for Drp1^9, 12, 13^. Despite they both function independently as receptors to bind and recruit cytosolic Drp1 to the mitochondrial surface, Mff and MIEFs have opposing effects on mitochondrial morphology following exogenous expression: overexpression of Mff results in excessive mitochondrial fragmentation^9, 14^, whereas overexpression of MIEF1 or MIEF2 leads to mitochondrial elongation most likely by inhibiting fission^11–13^. Thus, it is believed that Mff is the primary receptor for Drp1 to facilitate mitochondrial fission^9, 11, 14, 15^, whereas MIEFs recruit but presumably suppress Drp1’s function by sequestering the protein in an inactive state on the mitochondrial surface^11, 13, 16^. Although Mff, MIEF1 and MIEF2 as well as hFis1 are known to be simultaneously expressed in cells^17, 18^, it is unclear whether and how these receptors might work coordinately to regulate Drp1 recruitment to mitochondria. In addition, it has been difficult to understand why overexpression and depletion of MIEFs both result in a mitochondrial fusion phenotype^11–13, 18^. Therefore, how MIEFs are involved in regulating mitochondrial fission remains poorly understood.

In this report, it is shown that although Mff and MIEFs both are capable of serving as independent receptors for Drp1^9–11, 13, 16^, MIEFs can interact with both Drp1 and Mff, and thereby function as molecular adaptors linking Drp1 and Mff in a trimeric Drp1-MIEF-Mff complex on the surface of mitochondria. Furthermore, MIEFs regulate the association of Drp1 with Mff as well as Mff-induced Drp1 accumulation on mitochondria. In line with this, depletion of MIEF1/2 by siRNA treatment or by CRISPR/Cas9-based knockout impaired the physical association of Mff with Drp1, resulting in a decrease of Mff-induced Drp1 accumulation on mitochondria. In addition, we found that re-introduction of MIEF1 or MIEF2 into cells depleted of one or both MIEFs led to two distinct mitochondrial phenotypes dependent on the level of introduced MIEFs: in cells with lower levels of exogenous MIEFs, a mitochondrial fission phenotype was observed, whereas cells with higher levels of exogenous MIEFs displayed a fusion phenotype. Collectively, our data suggest that MIEFs and Mff can work coordinately in the process of Drp1-mediated fission in such a way that the levels of MIEF1/2 relative to Mff can set the balance between mitochondrial fission and fusion.

## Results

### MIEFs regulate Mff-mediated recruitment of Drp1 from the cytoplasm to mitochondria and affect Mff-induced Drp1 accumulation on mitochondria

**Figure 1:**
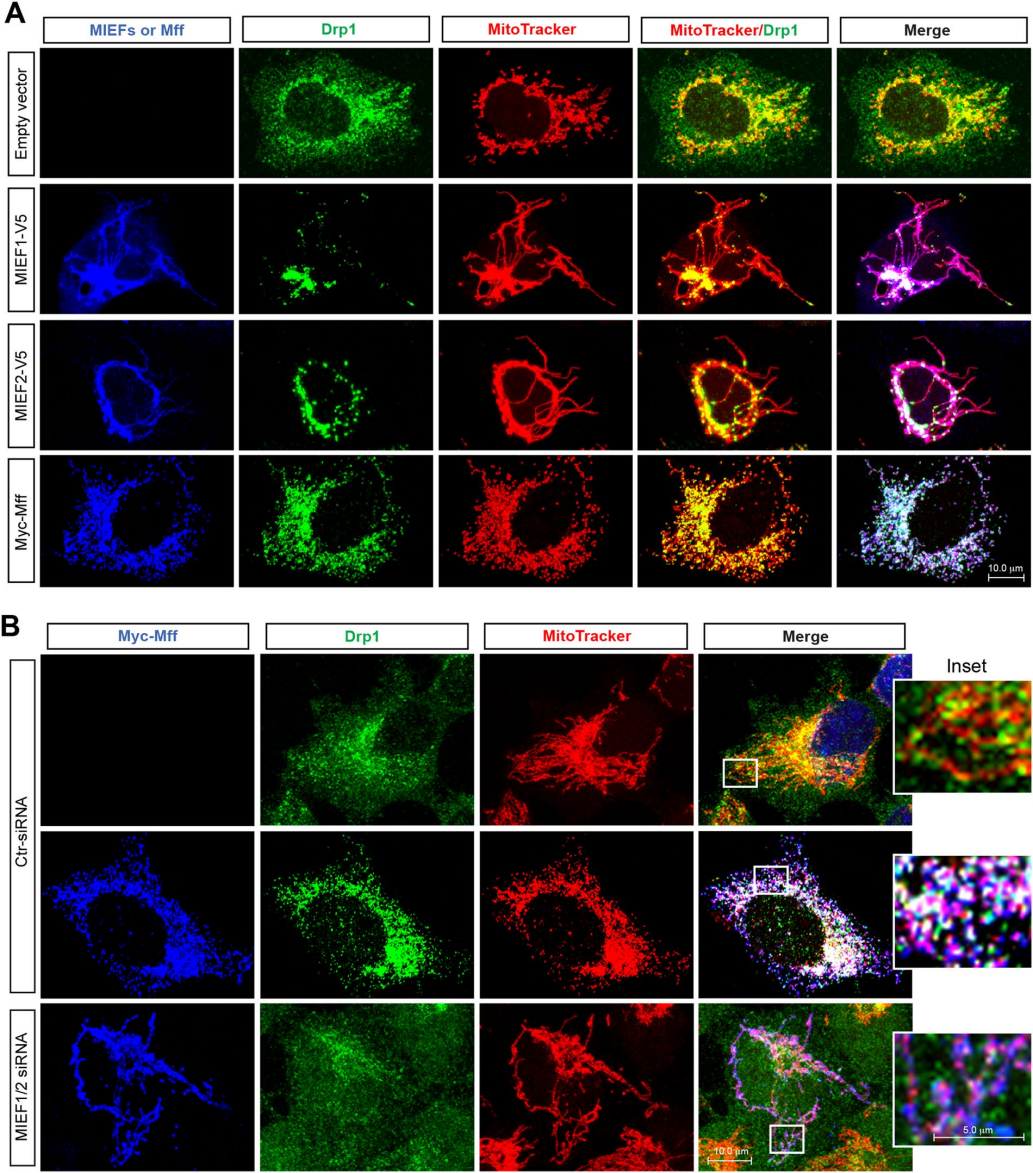
MIEFs and Mff recruit Drp1 to mitochondria, but have opposing effects on mitochondrial morphology. **(A)** Overexpression of either MIEF1, MIEF2 or Mff recruits Drp1 from the cytoplasm to mitochondria, but MIEF overexpression leads to a mitochondrial fusion phenotype, while Mff induces mitochondrial fission. Confocal images of mitochondrial morphology and Drp1 distribution in 293T cells transfected with indicated plasmids, stained with MitoTracker (red), anti-Drp1 (green), and either anti-V5 (blue for MIEF) or anti-Myc (blue for Mff) antibodies. **(B**) Confocal images of 293T cells treated with control siRNA or a combination of MIEF1 and MIEF2 siRNAs, followed by transfection with empty vector or Myc-Mff plasmid, and stained with MitoTracker (red), anti-Drp1 (green) and anti-Myc (blue) antibodies. Insets represent high magnification views of the boxed areas. Results from quantitative co-localization of Drp1 with mitochondria are summarized in Fig. 3D.

Mff and MIEF1/2 have emerged as key receptors for the recruitment of Drp1 to the MOM. It has been previously reported that simultaneous knockdown of MIEF1/2 (see Supplementary information, Figure S1A–S1C), or knockdown of Mff by siRNA treatment in both cases led to a significant decrease of Drp1 on mitochondria, resulting in mitochondrial elongation in 293T cells^9, 11–13, 19^. However, overexpression of MIEFs or Mff had opposing effects on mitochondrial dynamics: Overexpression of either MIEF1 or MIEF2 led to a mitochondrial fusion phenotype, whereas overexpression of Mff resulted in extensive mitochondrial fission (Fig. 1A). This suggests that Mff and MIEFs play distinct roles in Drp1-mediated mitochondrial fission.

To address the opposing effects of Mff and MIEFs on mitochondrial morphology, we first assessed whether MIEFs and Mff, in addition to that they serve as independent receptors for Drp1 recruitment to mitochondria, also can work together and coordinately regulate this recruitment. Interestingly, exogenous expression of Mff alone led to a significant accumulation of Drp1 on mitochondria in the presence of endogenous MIEF1/2 (Fig. 1B, middle panel), while simultaneous knockdown of endogenous MIEF1/2 by siRNA greatly reduced Mff-mediated Drp1 accumulation on mitochondria (Fig. 1B, lower panel, also summarized in Fig. 3D). This result suggests that endogenous MIEFs facilitate Mff-mediated recruitment of Drp1 to mitochondria.

**Figure 2:**
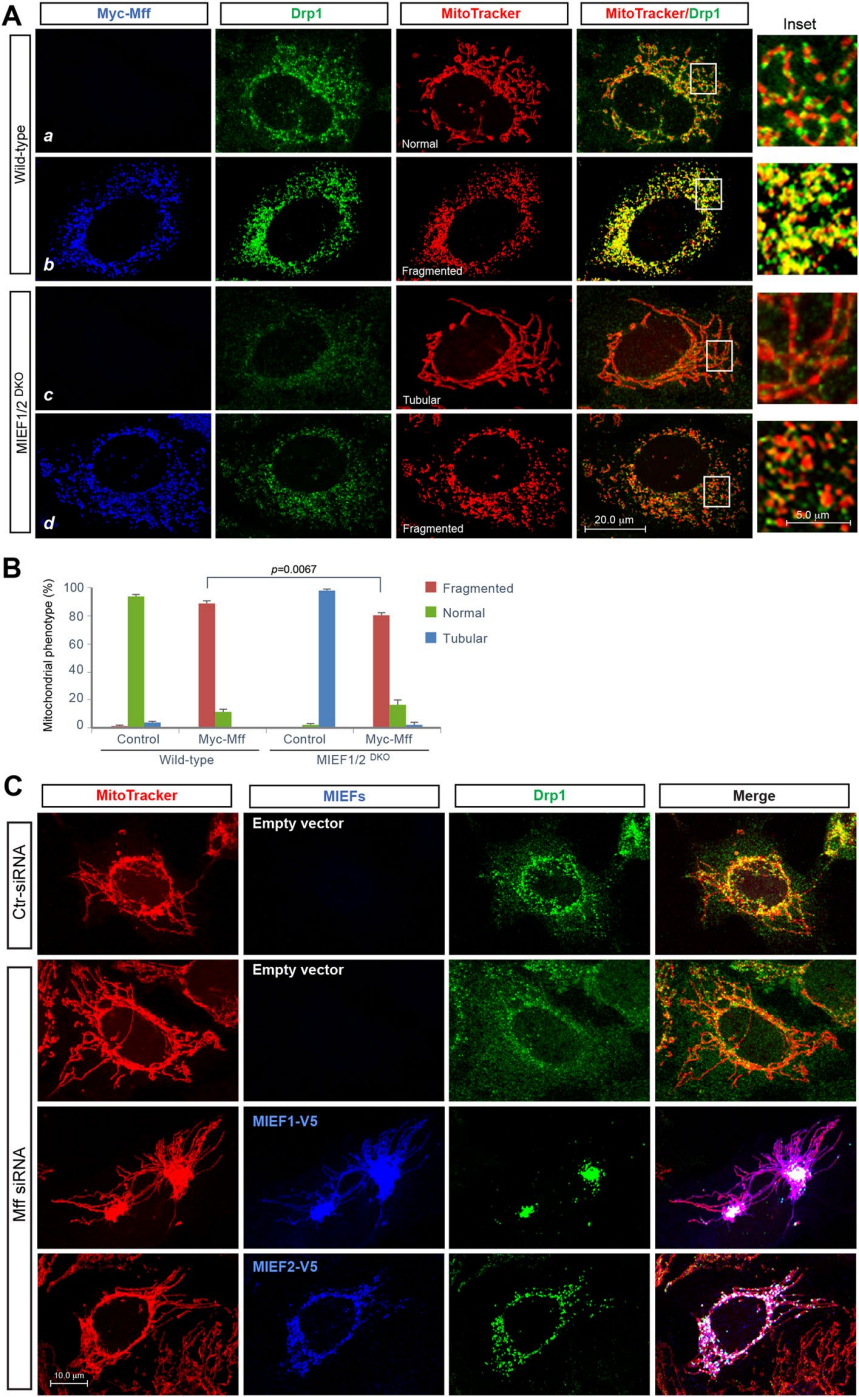
Ablation of MIEF1/2 impairs Mff-induced accumulation of Drp1 on mitochondria, whereas knockdown of Mff does not impair MIEF-mediated recruitment of Drp1 to mitochondria. (**A**) Confocal images of mitochondrial morphology and Drp1 distribution in wild-type and *MIEF1/2* ^DKO^ 293T cells transfected with empty vector or Myc-Mff plasmid. Cells were stained with MitoTracker (red) followed by immunostaining with anti-Drp1 (green) and anti-Myc (blue) antibodies. Insets represent high magnification views of the boxed areas. Quantitative co-localization of Drp1 with mitochondria is summarized in Fig. 3D. (**B**) Percentages (mean ± SEM) of cells with indicated mitochondrial morphologies in wild-type and *MIEF1/2* ^DKO^ 293T cells transfected with empty vector or Myc-Mff plasmid in three independent experiments (≥150 cells were analyzed per experiment). (**C**) Knockdown of Mff does not affect MIEF-mediated recruitment of Drp1 to mitochondria. Confocal images show Drp1 distribution on mitochondria and in the cytoplasm. 293T cells were treated with control siRNA, or Mff siRNA, followed by transfection with either empty vector or with MIEF1-V5 or MIEF2-V5 as indicated, and stained with MitoTracker (red), anti-Drp1 (green) and anti-V5 (blue) antibodies.

**Figure 3:**
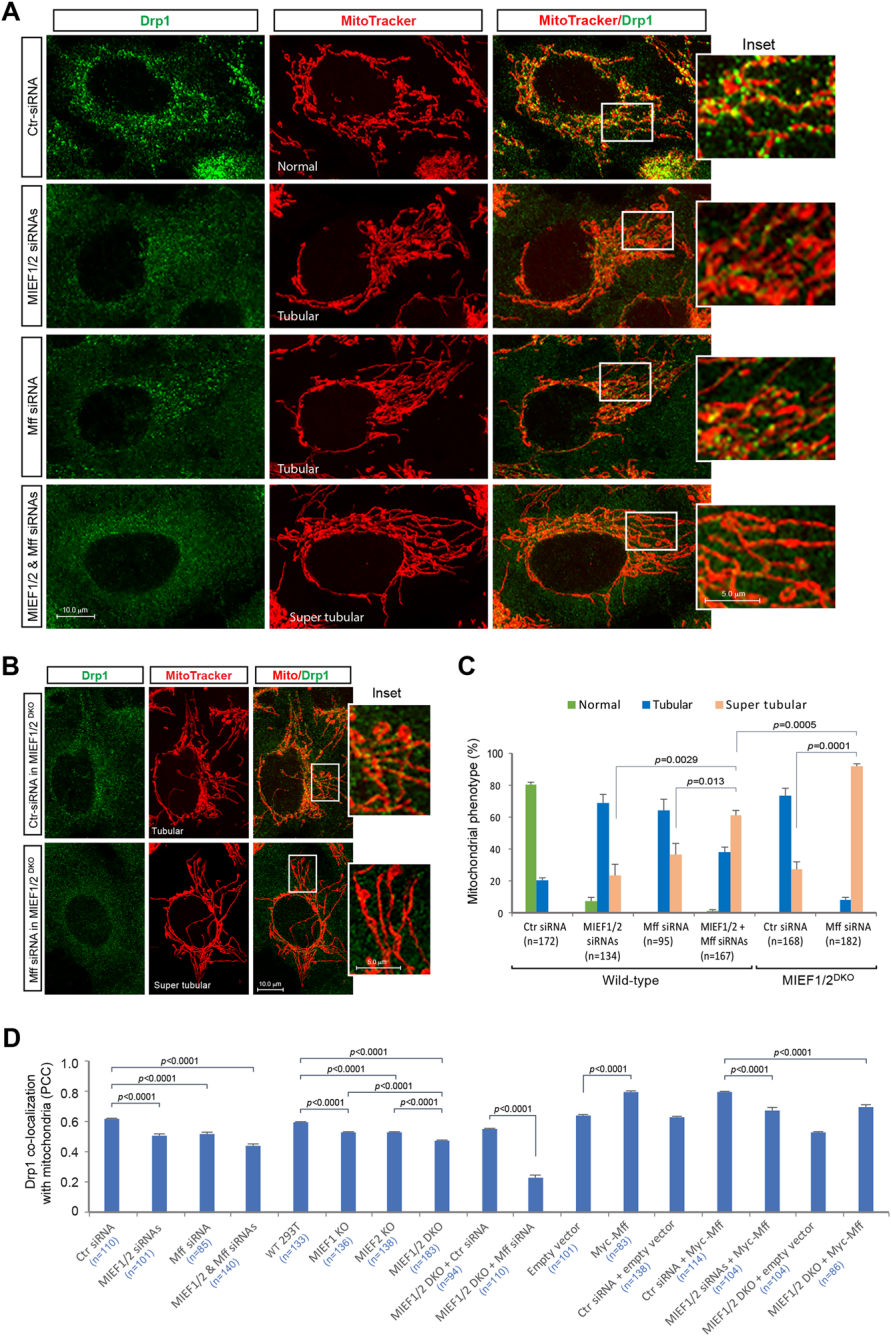
Triple ablation of MIEF1/2 and Mff significantly reduces the distribution of Drp1 on mitochondria, and results in a severe mitochondrial fusion phenotype. (**A**) Effects of triple knockdown of MIEF1/2 and Mff by siRNAs on mitochondrial shape and Drp1 distribution on mitochondria. Confocal images of mitochondrial morphology and Drp1 distribution in 293T cells treated with control siRNA, MIEF1/2 siRNAs, Mff siRNA, and MIEF1/2 siRNAs plus Mff siRNA, and stained with MitoTracker (red) followed by immunostaining with anti-Drp1 (green) antibody. Percentages (mean ± SEM) of cells with indicated mitochondrial morphologies are summarized in (**C**), and quantitative co-localization of Drp1 with mitochondria in different conditions is summarized in (**D**). (**B**) Effects of Mff knockdown by siRNA on mitochondrial shape and Drp1 distribution on mitochondria in the absence of MIEFs. Confocal images of mitochondrial morphology and Drp1 distribution in *MIEF1/2* ^DKO^ 293T cells treated with control siRNA or Mff siRNA, and stained as in (**A**). Quantitative co-localization of Drp1 with mitochondria is summarized in (**D**). (**C**) Percentages (mean ± SEM) of cells with indicated mitochondrial morphology in wild-type and *MIEF1/2* ^DKO^ 293T cells transfected with indicated siRNAs in three independent experiments (≥30 cells were analyzed per experiment). Here the tubular morphology of mitochondria is indicated as moderate (tubular) or “super tubular”. The latter cells display extensively long mitochondria. Total cell numbers (n) used for statistical analysis by the Student’s *t*-test are indicated in each condition. (**D**) Quantitative co-localization of endogenous Drp1 with mitochondria was analyzed using the Pearson’s correlation coefficient (PCC) (mean ± SEM) in different conditions as indicated. Each set of data is based on three independent experiments. Total cell numbers (n) used for statistical analysis are indicated for each condition.

To further evaluate the role of MIEFs in controlling Mff-mediated recruitment of Drp1 to mitochondria, we generated *MIEF1* and *MIEF2* single-knockouts (*MIEF1* ^KO^ and *MIEF2* ^KO^), as well as *MIEF1/2* double-knockout (*MIEF1/2* ^DKO^) 293T cell lines (Supplementary information, Figure S2) using CRISPR/Cas9 gene-editing technology^20^. As compared to wild-type controls, knockout of either MIEF1 or MIEF2 alone resulted in an obvious decrease of Drp1 on the mitochondrial surface (Supplementary information, Figure S3A, also summarized in Fig. 3D) and a moderate mitochondrial fusion phenotype in some cells (Supplementary information, Figure S3A and S3B). Double-knockout of MIEF1/2 induced further mitochondrial fusion, resulting in tubular networks of mitochondria in most cells (Supplementary information, Figure S3A and S3B), accompanied by a further decrease in the level of Drp1 on mitochondria (Supplementary information, Figure S3A, also summarized in Fig. 3D). In line with the results presented in Fig. 1B, Mff overexpression-induced Drp1 accumulation on mitochondria was significantly reduced in *MIEF1/2* ^DKO^ cells (Fig. 2A, panel *d*, compared to wild-type control in panel *b*, also summarized in Fig. 3D). In addition, we analyzed the effect of MIEF1/2 knockout on Mff-mediated mitochondrial fission. In *MIEF1/2* ^DKO^ 293T cells, although overexpression of Mff still stimulated extensive mitochondrial fragmentation in most of cells, loss of endogenous MIEF1/2 resulted in a decrease of cells with fragmented mitochondria, compared to wild-type controls (Fig. 2A and B), indicating that ablation of MIEF1/2 impairs Mff overexpression-induced mitochondrial fragmentation. Collectively, these results further support an important role for MIEFs in regulating Mff-mediated Drp1 recruitment to and Mff-induced Drp1 accumulation on mitochondria.

In contrast, we found that although knockdown of endogenous Mff alone by siRNA significantly reduced the levels of Drp1 on mitochondria (Fig. 2C, comparing upper two panels, also summarized in Fig. 3D), this did not impair MIEF overexpression-induced recruitment of Drp1 to the MOM (Fig. 2C, lower two panels), compared to controls (upper two panels) and compared to Drp1 recruitment in the presence of endogenous Mff (see Fig. 1A), in keeping with previous reports^11, 13, 16^. Together these data indicate that MIEFs recruit Drp1 to mitochondria in an Mff-independent manner, whereas the Mff-induced accumulation of Drp1 on the MOM partially depends on the presence of endogenous MIEFs. In conclusion, all results presented here argue that MIEFs can regulate Mff-mediated recruitment and accumulation of Drp1 on mitochondria.

We next examined the effects of triple ablation of MIEF1/2 and Mff by siRNAs on Drp1’s distribution on mitochondria and on mitochondrial morphology. We found that triple knockdown of MIEF1/2 and Mff by siRNAs had a more severe impact on both Drp1 distribution on mitochondria (Fig. 3A and D) and mitochondrial phenotype (Fig. 3A and C) respectively, compared to either MIEF1/2 or Mff knockdown alone. Similarly, knockdown of Mff by siRNA in *MIEF1/2* ^DKO^ cells resulted in a more severe decrease of Drp1 levels on mitochondria (Fig. 3B and D) and caused a more severe mitochondrial elongation phenotype (i.e. super tubular mitochondria) (Fig. 3B and C). Taken together, the data suggest that Mff and MIEFs all are able to recruit Drp1 to mitochondria independently as previously reported^10^, and implies that endogenous MIEFs and Mff might have additive effects on Drp1 accumulation on mitochondria, whereas the ability of Mff to induce Drp1 accumulation on mitochondria is obviously diminished in the absence of endogenous MIEFs.

To exclude that the genetic modifications, i.e. by overexpression, knockout or/and knockdown of MIEFs and Mff in cells had un-foreseen compensatory effects, we finally evaluated the levels of Drp1, MIEFs and Mff in the different conditions (Supplemental information, Figure S4A–S4F). Western blots revealed that overexpression of either MIEF1 or MIEF2 did not affect the levels of Drp1 or Mff (Figure S4A), and that overexpression of Mff had no discernible effects on Drp1, MIEF1 or MIEF2 (Figure S4B). Likewise, ablation of Mff by siRNA alone or in combination with either MIEF1 or MIEF2 overexpression (Figure S4C and S4D) had no major effects on cellular Drp1 levels. Further, we showed that knockout of MIEF1/2 alone (Figure S4E) or in combination with Mff overexpression (Figure S4F) had no discernable effects on total Drp1 levels in 293T cells.

**Figure 4:**
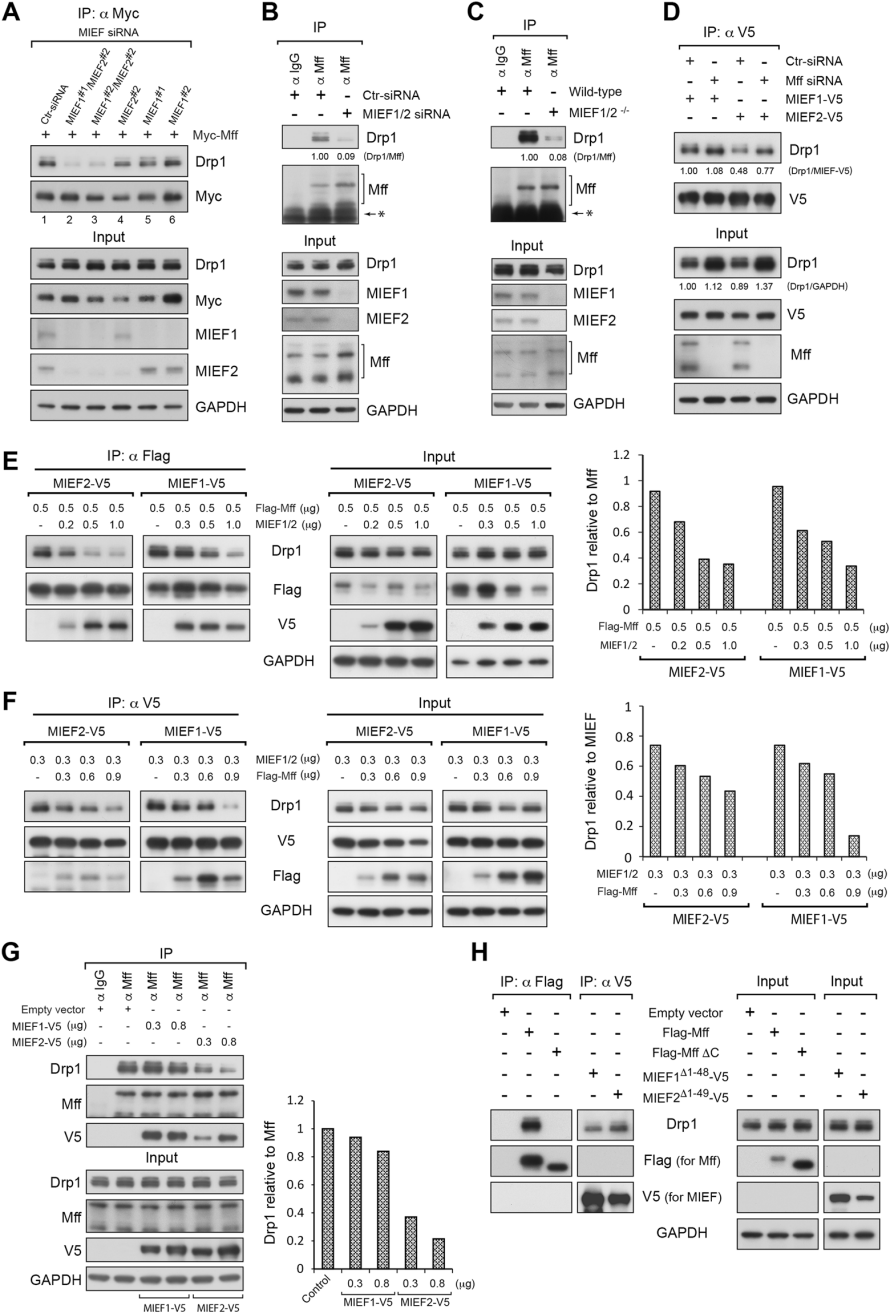
Depletion of MIEF1/2 largely reduces Mff-Drp1 interaction and the binding preference of Drp1 to MIEFs versus Mff depends on the relative amounts of MIEFs and Mff. (**A**) Knockdown of MIEF1/2 severely reduced the binding of exogenous Mff to Drp1. 293T cells were treated with control, MIEF1 or MIEF2 siRNA alone, or MIEF1 plus MIEF2 siRNAs in two different combinations, and then transfected with Myc-Mff. Cell lysates were used for co-IP with anti-Myc beads. (**B**) Knockdown of MIEF1/2 severely reduced the endogenous interaction between Mff and Drp1. 293T cells were treated with control, or MIEF1 plus MIEF2 siRNAs. Cell lysates were used for co-IP at endogenous levels with goat normal IgG (negative control) or goat anti-Mff antibody. The ratio of co-IPed endogenous Drp1/Mff was analyzed by densitometry. *Represents protein G. (**C**) Knockout of MIEF1/2 severely reduced endogenous interaction of Mff with Drp1. Cell lysates from wild-type or *MIEF1/2* ^DKO^ 293T cells were used for co-immunoprecipitation (IP) with Protein G beads connected to goat normal IgG (negative control) or goat anti-Mff antibody. The ratio of co-IPed endogenous Drp1/Mff was analyzed by densitometry. *Represents protein G. (**D**) Knockdown of Mff does not impair the association between MIEFs and Drp1. 293T cells were treated with control or Mff siRNA, and then transfected with MIEF1-V5 or MIEF2-V5. Cell lysates were used for co-IP with anti-V5 beads. The ratio of co-IPed Drp1/MIEF-V5 (IP) and total Drp1/GAPDH (Input) were analyzed by densitometry. The variation of Drp1 input signals between lanes is due to unequal protein loading (see also Fig. S4C and S4D). In (**A**–**D**), all co-IPs were analyzed by immunoblotting with indicated antibodies. (**E**) Elevated levels of MIEF1 or MIEF2 reduce the interaction of exogenous Flag-Mff with Drp1. 293T cells were co-transfected with Flag-Mff (0.5 µg) and either MIEF1-V5 or MIEF2-V5 in different amounts as indicated. Cell lysates were used for co-IP with anti-Flag beads followed by immunoblotting with indicated antibodies. The ratio of co-IPed Drp1/Mff is shown to the right. (**F**) Elevated levels of Mff reduce the MIEF-Drp1 interaction. 293T cells were co-transfected with 0.3 µg of either MIEF1-V5 or MIEF2-V5 and Flag-Mff in different amounts as indicated. The lysates were used for co-IP with anti-V5 agarose followed by immunoblotting with indicated antibodies. The ratio of co-IPed Drp1 to MIEF signal is shown to the right. (**G**) Elevated levels of MIEF1 or MIEF2 also reduce the interaction of endogenous Mff with Drp1. 293T cells were transfected with either MIEF1-V5 or MIEF2-V5 in different amounts as indicated. Cell lysates were used for co-IP with goat anti-Mff antibody followed by immunoblotting with indicated antibodies. Goat normal IgG was used as the negative control. The ratio of co-IPed Drp1/Mff is shown to the right. (**H**) Mitochondrial localization is required for the association of Mff with Drp1 but not for MIEFs to associate with Drp1. 293T cells were transfected with wild-type Flag-Mff, or transfected with the cytoplasmic mutants Flag-Mff∆C, MIEF1^Δ1–48^ or MIEF2^Δ1–49^. Cell lysates were used for co-IP with anti-Flag (for Mff) and anti-V5 (for MIEF) beads followed by immunoblotting with indicated antibodies.

### MIEFs orchestrate the interaction between Mff and Drp1

We subsequently evaluated whether depletion of MIEFs affected the association between Mff and Drp1. In cells with endogenous MIEF1/2 or with only one of the MIEFs knocked down by siRNA, Mff interacted with Drp1 (Fig. 4A, lanes 1, 4–6). However, simultaneous knockdown of MIEF1/2 substantially decreased the association of exogenous Mff with Drp1 (Fig. 4A, lanes 2, 3). Importantly, at endogenous levels, co-IP using antibodies against Mff revealed that both knockdown (Fig. 4B) and knockout (Fig. 4C) of MIEF1/2 severely reduced Mff-Drp1 interaction, confirming that MIEFs are crucial for maintaining the binding of Mff to Drp1. Conversely, when Mff was knocked-down by siRNA, MIEFs still interacted with Drp1 normally (Fig. 4D), suggesting that ablation of Mff had no evident impact on the interaction between MIEFs and Drp1. Collectively, the results further support that MIEFs can facilitate the interaction between Mff and Drp1, whereas Mff does not affect the binding of MIEFs to Drp1.

We next asked how different levels of MIEFs or Mff influenced the association of Drp1 with Mff or MIEFs, respectively. Interestingly, we found that elevated levels of either MIEF1 or MIEF2 (especially MIEF2) reduced the co-precipitation of both exogenous Mff (Fig. 4E) and endogenous Mff (Fig. 4G) with Drp1 in a dose-dependent manner. Conversely, increased expression of Mff reduced the association of MIEF1/2 with Drp1 in a dose-dependent manner (Fig. 4F). Together, these co-IP experiments show that MIEFs and Mff compete for the interaction with Drp1, thereby potentially impacting on the balance of mitochondrial dynamics as previously reported^19^.

We next explored whether mitochondrial localization of MIEFs and Mff was a prerequisite for their association with Drp1. Co-immunoprecipitation (co-IP) showed that the cytoplasmic mutant Mff∆C, lacking the C-terminus and TM domain^9^, failed to bind Drp1 (Fig. 4H). In contrast, the cytoplasmic mutants MIEF1^Δ1–48^ and MIEF2^Δ1–49^, which lack the respective N-terminus and TM domain^13, 19^, retained Drp1 binding (Fig. 4H). This indicates that integration in the mitochondrial membrane is required for Mff, but not for MIEF to interact with Drp1, which suggests that MIEFs and Mff are functionally different in their interaction with/binding to Drp1 as well as in their potential to recruit Drp1 from the cytoplasm to mitochondria.

### MIEFs interact with both Mff and Drp1 to form a trimeric protein complex on the mitochondrial surface

**Figure 5:**
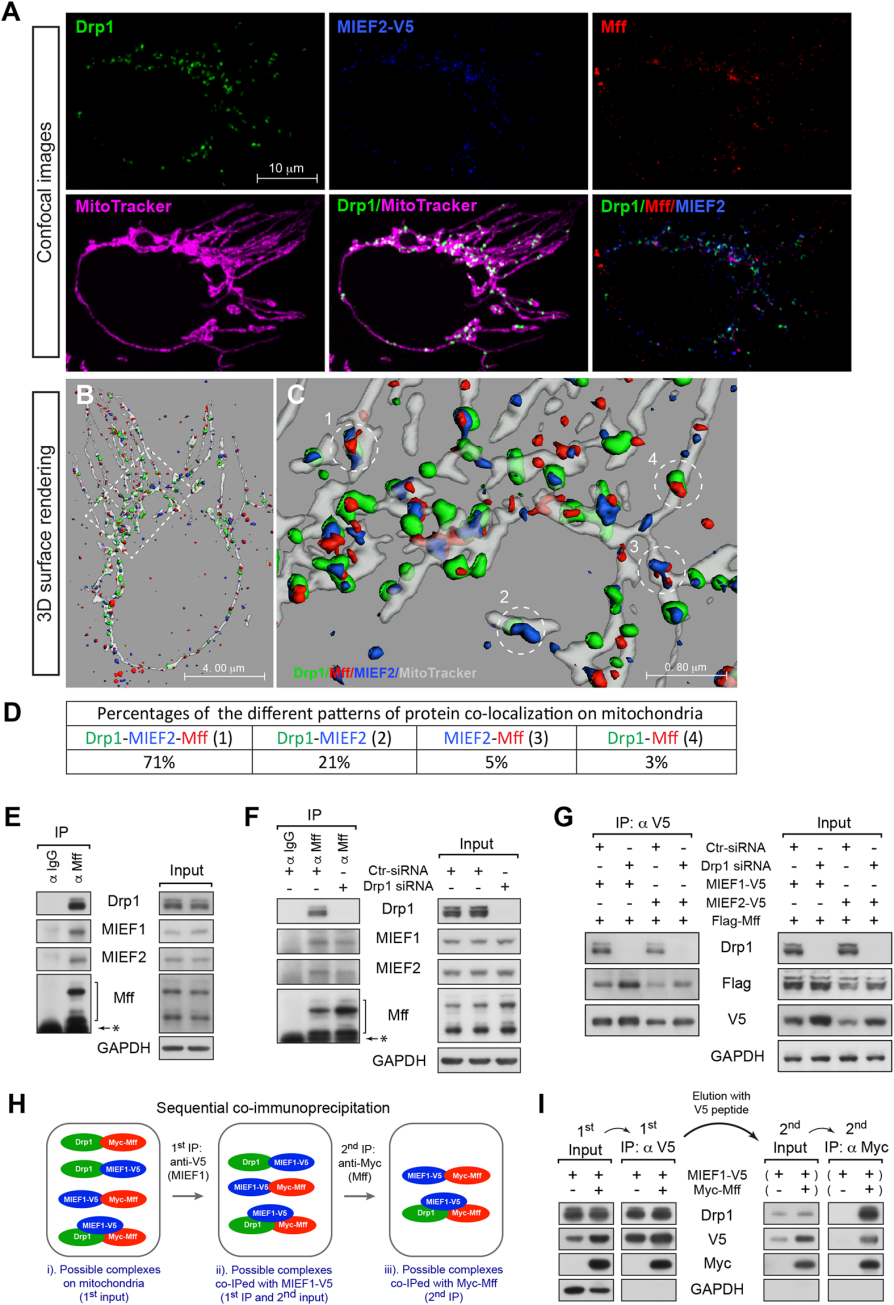
MIEF and Mff interact and form a trimeric complex with Drp1 on the mitochondrial surface. (**A**) Confocal images of 293T cells with stable expression of MIEF2-V5, stained with MitoTracker (red), followed by immunofluorescence staining with anti-V5 for MIEF2 (blue), anti-Drp1 (green) and anti-Mff (red) antibodies. (**B**) Surface rendered three-dimensional reconstructions of the cell as shown in (**A**). The MIEF2 (blue), Drp1 (green) and Mff (red) proteins, as well as mitochondria (gray) are indicated. (**C**) A high magnification view of the boxed area in (**B**) shows the different patterns of protein co-localization on mitochondria, including Drp1-MIEF2-Mff (1), Drp1-MIEF2 (2), MIEF2-Mff (3) and Drp1-Mff (4) as indicated by numbers. (**D**) Percentages of the different protein co-localization patterns on mitochondria as observed in (**C**). The data were obtained from 3D surface rendering images of mitochondria in two cells. (**E**) Mff interacts with MIEF1, MIEF2 and Drp1 at endogenous levels. Lysates from 293T cells were used for co-IP with either goat normal IgG or goat anti-Mff antibody followed by immunoblotting with indicated antibodies. *Represents protein G. (**F**) Knockdown of Drp1 does not affect the endogenous MIEF-Mff association. 293T cells were treated with either control or Drp1 siRNA. Cell lysates were used for co-IP with either goat normal IgG or goat anti-Mff antibody followed by immunoblotting with indicated antibodies. *Represents protein G. (**G**) Knockdown of Drp1 does not affect the exogenous MIEF-Mff interaction. 293T cells were transfected with control or Drp1 siRNA, and then co-transfected with Flag-Mff and either MIEF1-V5 or MIEF2-V5 plasmids. Cell lysates were used for co-IP with anti-V5 beads followed by immunoblotting with indicated antibodies. (**H**) Outline of sequential co-immunoprecipitation (co-IP) experiments designed in (**I**) to determine whether a trimeric Drp1-MIEF-Mff complex existed on mitochondria in cells. (**I**) MIEF, Mff and Drp1 interact in a trimeric protein complex. 293T cells were co-transfected with MIEF1-V5 and Myc-Mff. Cell lysates were used for sequential co-IP: the first (1^st^) co-IP was performed with anti-V5 beads, and then MIEF1-V5 and its associated proteins were eluted with V5 peptide and used for a second (2^nd^) co-IP with anti-Myc beads. The 1^st^ and 2^nd^ rounds of co-IPed proteins were analyzed by immunoblotting with indicated antibodies.

Because MIEFs as shown above can regulate the interaction between Mff and Drp1, we further explored the relationship of MIEFs with Drp1 and Mff. To this end, we first examined the co-localization of MIEFs with Drp1 and Mff on mitochondria in a 293T cell line with stable expression of MIEF2-V5, i.e. a level sufficient to induce a significant accumulation of Drp1 at the mitochondrial surface, accompanied by a moderate mitochondrial fusion phenotype (Fig. 5A). Confocal microscopy combined with 3D surface rendering revealed four different forms of co-localization at the mitochondrial surface: three dimeric forms (Drp1-MIEF2, MIEF2-Mff and Drp1-Mff) and the trimeric form (Drp1-MIEF2-Mff) (Fig. 5B and C). The trimeric form (Drp1-MIEF2-Mff) was most abundant (~71%), while colocalization between only two of the proteins was considerably less frequent (MIEF2-Drp1, MIEF2-Mff and Drp1-Mff were found in ~21%, ~5% and 3% respectively of analyzed colocalization sites at the MOM) in these cells with a moderate mitochondrial fusion phenotype (Fig. 5D). The existence of the dimeric Drp1-MIEF2 and Drp1-Mff complexes was corroborated by the co-IP experiments shown in Fig. 4B–D, and co-IP for Mff revealed that both MIEF1/2 and Drp1 were co-precipitated with Mff at endogenous protein levels (Fig. 5E). Furthermore, knockdown of Drp1 by siRNA did not disrupt the endogenous interaction between MIEFs and Mff (Fig. 5F), and neither did it disrupt the association of exogenous MIEF1-V5 or MIEF2-V5 with Flag-Mff (Fig. 5G), suggesting that the MIEF-Mff interaction can occur independently of Drp1.

To further corroborate the presence of a trimeric protein complex containing MIEF, Drp1 and Mff, we designed a sequential co-IP experiment as illustrated in Fig. 5H: a first pulldown by MIEF1 (anti-V5), followed by a second pulldown by Mff (anti-Myc). Given the existence of four different protein complexes on mitochondria as described above (Fig. 5A–D, see also Fig. 5H, i), the Drp1-Mff complex should be lost in the first co-IP with anti-V5 beads (for MIEF1-V5), whereas the other three complexes (i.e. MIEF1-Drp1, MIEF1-Mff and a possible trimeric complex of MIEF1, Drp1 and Mff) should be co-IPed with anti-V5 beads (Fig. 5H, ii). Subsequently, MIEF1-V5 and associated complexes are eluted from the anti-V5 beads using V5 peptide and subjected to a second co-IP using anti-Myc beads, by which the Drp1-MIEF1-V5 complex is eliminated, whereas the MIEF1-V5-Myc-Mff complex is still retained in this second co-precipitate. Drp1 should also be detected in the second co-precipitate if a trimeric Drp1-MIEF-Mff complex exists (Fig. 5H, iii). If not, the trimeric complex does not exist on mitochondria. To test this, 293T cells were co-transfected with MIEF1-V5 and Myc-Mff and subjected to the sequential co-IPs (Fig. 5I). Immunoblot analysis revealed the presence of MIEF1, Mff and Drp1 following the second co-IP (Fig. 5I, right panel), indicating that the three proteins indeed form a trimeric complex in cells.

### MIEFs serve as adaptors linking Drp1 with Mff in a trimeric Drp1-MIEF-Mff complex

**Figure 6:**
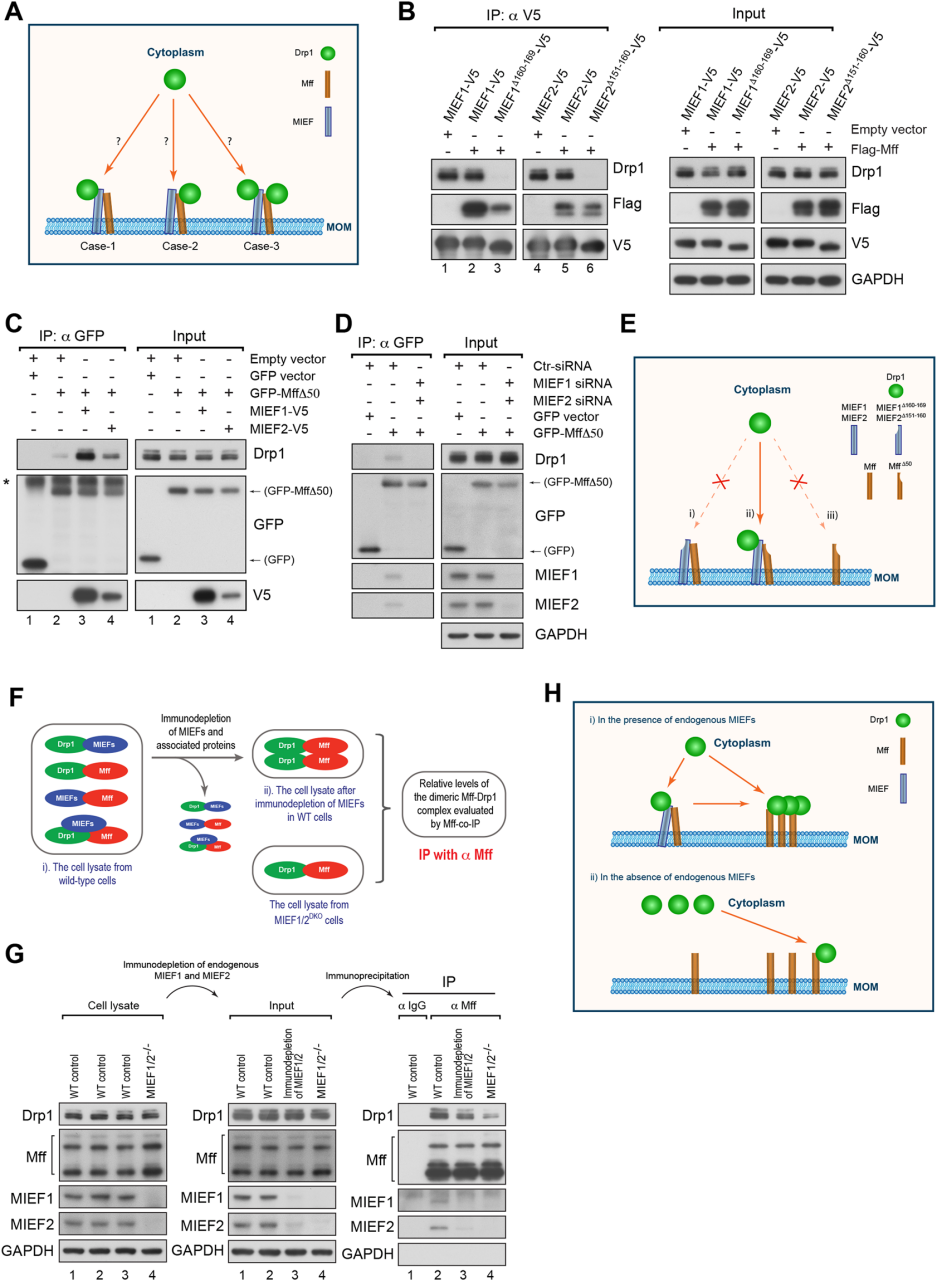
MIEF acts as an adaptor linking Drp1 and Mff in a trimeric Drp1-MIEF-Mff complex. (**A**) A schematic diagram for three possible Drp1 assembly modes in a trimeric Drp1-MIEF-Mff complex on mitochondria. Case-1: Drp1 binds only to MIEF; Case-2: Drp1 binds only to Mff; Case-3: Drp1 binds to both MIEF and Mff. MOM: mitochondrial outer membrane. (**B**) Drp1 is unable to bind Mff in the Drp1-MIEF-Mff complex. 293T cells were co-transfected with indicated plasmids. MIEF1^Δ160–169^ and MIEF2^Δ151–160^ represent Drp1 binding-deficient MIEF mutants. Cell lysates were used for co-IP with anti-V5 beads followed by immunoblotting with indicated antibodies. (**C**) Drp1 interacts with MIEF in the trimeric Drp1-MIEF-Mff complex. 293T cells were co-transfected with empty vector and GFP vector, or with GFP-Mff∆50 (a Drp1 binding-deficient Mff mutant) together with empty vector, MIEF1-V5 or MIEF2-V5 plasmid as indicated. Cell lysates were used for co-IP with anti-GFP beads followed by immunoblotting with indicated antibodies. *Represents IgG heavy chain. (**D**) Drp1 is brought to the Mff∆50-associated complex via endogenous MIEF. 293T cells were treated with control, or MIEF1 plus MIEF2 siRNAs, and then transfected with GFP-vector or with GFP-Mff∆50 plasmid as indicated. Cell lysates were used for co-IP with anti-GFP beads followed by immunoblotting with indicated antibodies. (**E**) Schematic diagram for illustrating the experimental results obtained in (**B**–**D**). These results demonstrated that MIEFs act as adapters linking Drp1 and Mff together in a trimeric Drp1-MIEF-Mff complex. (i) represents the results in (**B**); (ii) represents the results in (**C**); (iii) represents the results in (**D**). MOM: mitochondrial outer membrane. (**F**) A schematic diagram to illustrate the strategy of the experiment performed in (**G**). To compare the levels of the dimeric Mff-Drp1 complex in the presence (i.e. in wild-type cells) and absence (i.e. in *MIEF1/2* ^DKO^ cells) of endogenous MIEFs by co-IP. In wild-type cells MIEFs and MIEFs-associated proteins complexes (including the trimeric Drp1-MIEF-Mff complex) must be removed from the cell lysate by immunoprecipitation with anti- MIEF1 and MIEF2 antibodies before co-IP with Mff, and then Mff co-IP is performed in parallel with the cell lysate from *MIEF1/2* ^DKO^ cells as illustrated. (**G**) Absence of endogenous MIEFs markedly reduced levels of the dimeric Mff-Drp1 complex. Left panel: Western blots of cell lysates from wild-type and *MIEF1/2* ^*DKO*^ 293T cells as indicated. Middle panel: Western blots from the resulting supernatants (Input) after immunodepletion of endogenous MIEFs (see lane 3). For this immunodepletion, the cell lysate from wild-type 293T cells (lane 3) was incubated with Dynabeads® protein G conjugated with rabbit MIEF1- and MIEF2-specific antibodies (lane 3) and other cell lysates were incubated with Dynabeads® protein G conjugated with rabbit normal IgG as control (lanes 1, 2 and 4). Right panel: Western blots for co-IPs with Dynabeads® protein G conjugated with goat normal IgG or goat anti-Mff antibody followed by immunoblotting with indicated antibodies. Lane 1: negative control; Lane 2: positive control; Lane 3: wild-type cells after immunodepletion of MIEFs complexes; Lane 4: *MIEF1/2* ^DKO^ cells. (**H**) A schematic diagram illustrating the levels of the dimeric Mff-Drp1 complex in wild-type (i) and *MIEF1/2* ^DKO^ cells (ii) (i.e. in the presence and absence of endogenous MIEFs) from data presented in (**G**). MOM: mitochondrial outer membrane.

To obtain further insights into the organization of the protein complex containing MIEF, Mff and Drp1, we analyzed whether MIEF or Mff, or both, served as the interaction partner for Drp1 in the trimeric complex as illustrated in Fig. 6A. To address this issue, we used MIEFs and Mff deletion mutants, engineered not to interact with Drp1 but retain the MIEF-Mff interaction capacity. Co-expression of wild-type Mff with the Drp1 binding-deficient mutants MIEF1^Δ160–169^ or MIEF2^Δ151–160^ ^13, 19^ followed by co-IP for V5-tagged MIEFs revealed that both MIEF mutants interacted with Mff and a significant amount of Mff was co-immunoprecipitated with mutant MIEFs, whereas Drp1 was almost not detected in these complexes (Fig. 6B, lanes 3 and 6), indicating that Mff, when bound to MIEF, was not simultaneously bound to Drp1 (illustrated in Fig. 6E, i). This experiment suggests that the trimeric complex Case-1, but not Case-2 or Case-3 (illustrated in Fig. 6A) most likely exists in cells. To further explore this issue, we used the Mff deletion mutant GFP-Mff∆50, lacking the first 1–50 residues, which are important for Mff’s interaction with Drp1^9^. Interestingly, immunoprecipitation of GFP-tagged Mff∆50 revealed that a minute amount of Drp1 was co-precipitated with Mff∆50 in the presence of endogenous MIEFs (Fig. 6C, lane 2), and elevated levels of MIEFs by exogenous expression of either MIEF1-V5 or MIEF2-V5 resulted in an additional amount of Drp1 co-precipitated with Mff∆50 (Fig. 6C, lanes 3 and 4, compared to lane 2). This implies that Drp1 is likely brought into the trimeric complex via MIEFs (illustrated in Fig. 6E, ii). In line with this, Drp1 was no longer observed in the Mff∆50 immunoprecipitate after simultaneous knockdown of endogenous MIEF1/2 by siRNA (Fig. 6D, illustrated in Fig. 6E, iii). In summary, these data reveal the existence of a trimeric Drp1-MIEF-Mff complex, in which MIEFs interact with both Drp1 and Mff and therefore functions as adaptors linking Drp1 with Mff.

### Endogenous MIEFs facilitate a direct binding of Drp1 to Mff

As described above, MIEFs act as adaptors linking Drp1 and Mff in a trimeric Drp1-MIEF-Mff complex, thereby facilitating an indirect interaction between Mff and Drp1 by MIEFs forming a molecular bridge between Mff and Drp1. These findings prompted us to explore whether MIEFs were able to further promote a direct binding of Drp1 to Mff potentially via the trimeric complex. We therefore designed a set of experiments as illustrated in Fig. 6F to compare levels of the dimeric Drp1-Mff complex in the presence (i.e. in wild-type cells) and absence (i.e. in *MIEF1/2* ^DKO^ cells) of endogenous MIEFs by co-IP. As illustrated in the schematic diagram (Fig. 6F), several MIEFs-associated protein complexes (including the trimeric Drp1-MIEF-Mff complex) were simultaneously present in wild-type cells, while only the dimeric Mff-Drp1 complex was present in *MIEF1/2* ^DKO^ cells. To evaluate levels of the dimeric Mff-Drp1 complex in wild-type 293T cells, MIEFs and MIEF-associated proteins (including the trimeric Drp1-MIEF-Mff complex) were first immunodepleted from the cell lysate by MIEF1- and MIEF2-specific antibodies before co-IP with Mff (see also Fig. 6G, Input: lane 3 after immunodepletion of MIEFs and MIEFs-associated proteins), and then the cell lysate immunodepleted of MIEFs complexes was, in parallel with cell lysates from wild-type and *MIEF1/2* ^DKO^ 293T cells, co-immunoprecipitated with anti-Mff antibody. Immunoblotting showed that levels of the dimeric Drp1-Mff complex in *MIEF1/2* ^DKO^ cells were much lower than those in wild-type cells (Fig. 6G, IP, *MIEF1/2* ^DKO^ in lane 4 compared to wild-type in lane 3, see also Fig. 6H, ii), indicating that absence of endogenous MIEFs markedly reduced the amount of the dimeric Drp1-Mff complex and that endogenous MIEFs thus can promote the physical binding of Drp1 to Mff. In summary, our results support the idea that endogenous MIEFs recruit cytosolic Drp1 to mitochondria to form a trimeric Drp1-MIEF-Mff complex and facilitate a direct binding of Drp1 to Mff possibly via reassembly of Drp1 from the trimeric complex to a dimeric Drp1-Mff complex (Fig. 6H, i).

### MIEF1/2 levels regulate the balance between mitochondrial fission and fusion

**Figure 7:**
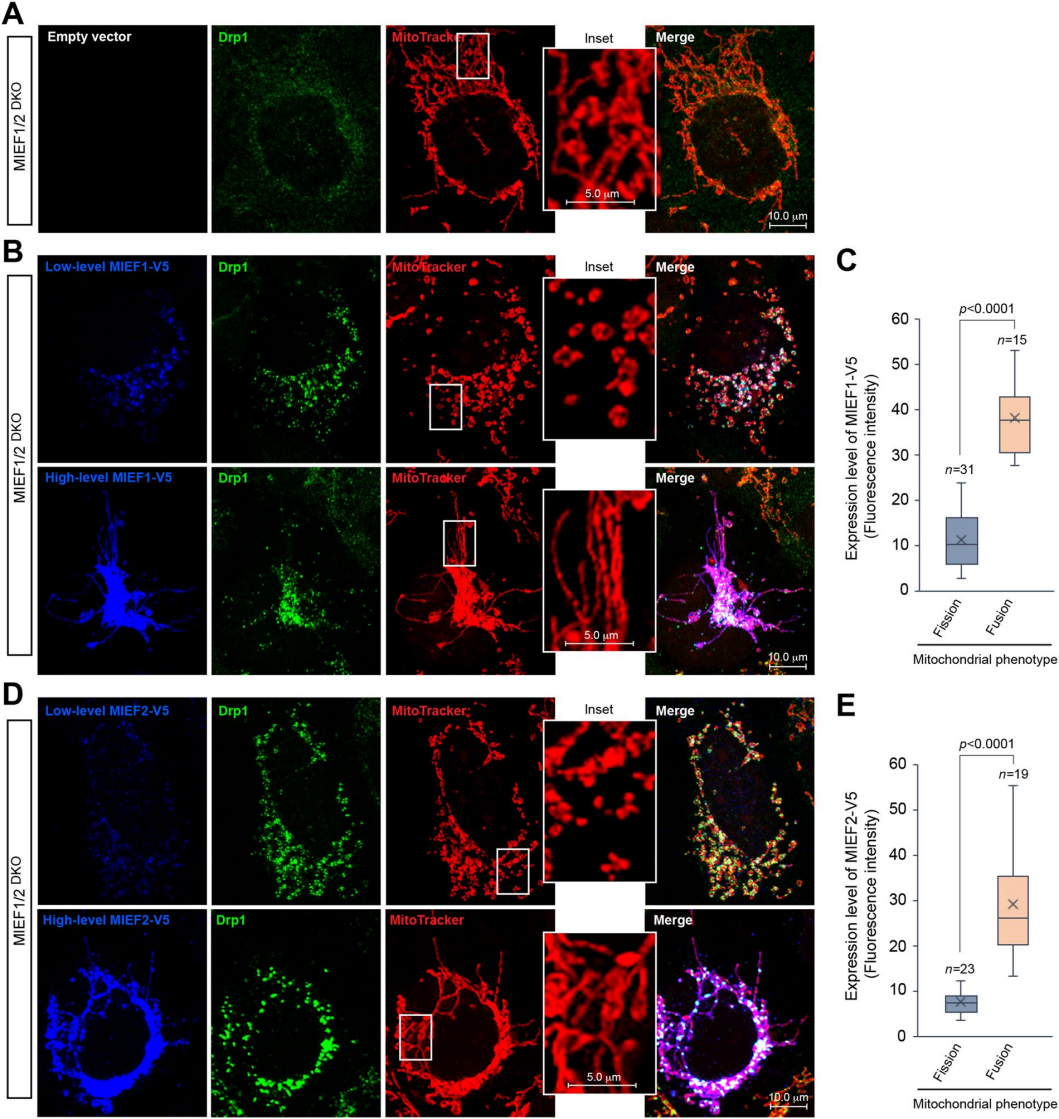
Different levels of MIEFs positively or negatively regulate mitochondrial fission. (**A**) Confocal images of mitochondrial morphology and Drp1 distribution in *MIEF1/2* ^*DKO*^ 293T cells transfected with empty vector as control. (**B**,**D**) Confocal images of mitochondrial morphology and Drp1 distribution in *MIEF1/2* ^*DKO*^ 293T cells transfected with MIEF1-V5 (**B**) or MIEF2-V5 (**D**) plasmid, stained with MitoTracker (red) followed by immunostaining with anti-Drp1 (green) and anti-V5 (blue) antibodies. Insets represent a magnified view of the respective boxed areas. (**C**,**E**) Box plots showing protein levels of MIEF1-V5 or MIEF2-V5 relative to the mitochondrial fission and fusion phenotypes. Expression levels of exogenous MIEF1-V5 (**C**) and MIEF2-V5 (**E**) in sets of cells as indicated showing either a mitochondrial fission or fusion phenotype were measured as immunofluorescence intensity using the Leica confocal microscopy software and shown by the box-plot analyses. The *p*-value represents the result from the Student’s t-test analysis.

It is known that depletion and overexpression of MIEFs both lead to a mitochondrial fusion phenotype, and only at low to intermediate levels are MIEFs able to maintain normal mitochondrial morphology. This prompted us to explore whether different levels of MIEFs could promote or inhibit mitochondrial fission. To this end, we re-introduced MIEF1 or MIEF2 into *MIEF1/2* ^DKO^ 293T cells and examined the mitochondrial morphology by immunofluorescence confocal microscopy. In *MIEF1/2* ^DKO^ cells (control), mitochondria exhibited a tubular network fusion phenotype (Fig. 7A). Interestingly, exogenous expression of either MIEF1 or MIEF2 in *MIEF1/2* ^DKO^ cells recruited cytosolic Drp1 to mitochondria, but both cases led to two distinct mitochondrial phenotypes depending upon the relative cellular levels of MIEFs: In general, cells with a mitochondrial fission phenotype had relatively lower levels of exogenous MIEF1- or MIEF2-V5 (Fig. 7B and D, upper panels), whereas cells with a fusion phenotype had relatively higher levels of exogenous MIEF1- or MIEF2-V5 (Fig. 7B and D, lower panels). The levels of exogenous MIEF1- and MIEF2-V5 relative to the mitochondrial phenotypes were measured by immunofluorescence intensity using the Leica confocal microscopy software and results are presented in Fig. 7C and E, respectively.

To further assess how the levels of MIEF1/2 potentially regulate the balance between mitochondrial fission and fusion, another set of experiments were performed: we first depleted MIEF1 by siRNA treatment of human 293T cells, and then transfected mouse *Mief1* cDNA (which is resistant to siRNAs designed for knockdown of the human MIEF1) back to restore expression. Exogenous mouse MIEF1 (mMIEF1) led to two distinct mitochondrial phenotypes depending on the relative mMIEF1 level: in cells with lower levels of exogenous mMIEF1, a fission phenotype was observed, whereas cells with higher levels of mMIEF1 showed a fusion phenotype (Supplementary information, Figure S5A, summarized in the right panel). Similar results were observed when mMIEF2 was introduced into 293T cells depleted of endogenous MIEF2 (Supplementary information, Figure S5B, summarized in the right panel). However, when mouse Mff (mMff) was introduced in 293T cells depleted of endogenous Mff, a fission phenotype was observed irrespective of low or high levels of transfected mMff (Supplementary information, Figure S5C). Taken together, these results support the notion that the relative abundance of MIEFs in cells fine-tune the mitochondrial fission activity, i.e. low-to-moderate levels of MIEFs promote mitochondrial fission, while high levels of MIEFs inhibit fission.

## Discussion

Drp1 recruitment to mitochondria is an essential step in mitochondrial fission. In addition to Fis1, Mff and MIEFs have been identified as receptors for the recruitment of Drp1 to the MOM in mammalian cells^9, 12, 13, 19^. While several studies have shown that Mff, MIEF1 and MIEF2 can serve as independent receptors to recruit Drp1 to mitochondria^10, 11, 16^, it is unclear if and how these Drp1 receptors work together to regulate Drp1-mediated fission activity. In a previous report, we showed that MIEF1 is able to either bind to Drp1 or to hFis1 in a mutually exclusive manner. When hFis1 interacts with MIEF1 to form an hFis1-MIEF1 protein complex, this sequesters MIEF1 so that it cannot bind to Drp1 anymore^13^. Consistently, overexpression of hFis1 reduces the amount of MIEF1/2 bound to Drp1 and this partially reverts the MIEF-induced mitochondrial fusion phenotype^19^.

**Figure 8:**
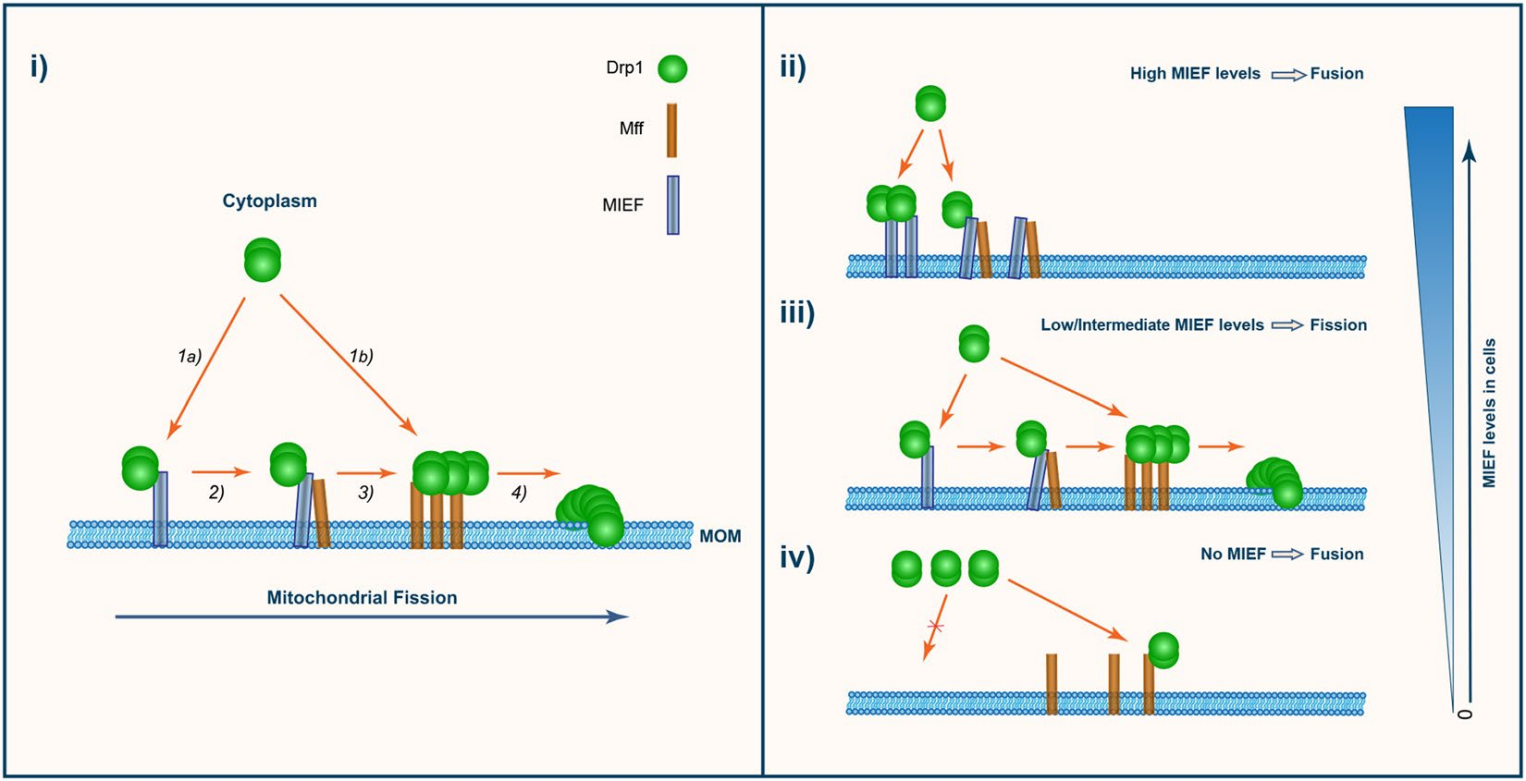
MIEF and Mff coordinately work in Drp1-mediated mitochondrial fission. (**i)** A working model illustrating how Drp1-mediated mitochondrial fission is regulated by a sequential and coordinated interaction of MIEF and Mff with Drp1. (1) At the initial step of fission, Drp1 is recruited by MIEF (1a) and also by Mff (1b) from the cytosol to the mitochondrial surface. (2) At the mitochondrial surface, MIEF serves as an adaptor linking Drp1 and Mff together in a trimeric Drp1-MIEF-Mff complex. (3) MIEF promotes a direct binding of Drp1 to Mff possibly via reassembly of Drp1 from the trimeric complex to a functional dimeric Drp1-Mff complex. (4) Drp1-mediated fission is activated, resulting in mitochondrial division. MOM: mitochondrial outer membrane. (**ii**–**iv**) The model depicts the outcome of different cellular levels of MIEF: (**ii)** High MIEF levels inhibit fission, leading to a fusion phenotype by sequestering Drp1 in a dimeric Drp1-MIEF or trimeric Drp1-MIEF-Mff complex. (**iii**) Low/intermediate MIEF levels promote fission by facilitating a direct binding of Drp1 to Mff. (**iv**) In the absence of MIEFs, Drp1 is not effectively recruited to the MOM via MIEFs with the result that Mff itself cannot capture sufficient amount of Drp1. As a consequence, the balance of mitochondrial dynamics is shifted towards fusion, resulting in a moderate mitochondrial elongation phenotype.

In this report, we show that MIEFs and Mff can act coordinately in the process of Drp1-mediated fission, in addition to that they serve as independent receptors to recruit Drp1 to mitochondria as previously reported^10, 11, 16^. The results presented here allow us to suggest a working model (Fig. 8, i), in which Drp1 can be partially recruited to mitochondria by Mff to activate Drp1-mediated fission, while MIEFs can recruit additional Drp1 to mitochondria and serve as adaptors linking Drp1 and Mff together in a trimeric Drp1-MIEF-Mff complex at the MOM. It is shown that MIEFs at physiological levels facilitate a direct binding of Drp1 to Mff, which is proposed to be followed by reassembly of Drp1 from the trimeric Drp1-MIEF-Mff complex to an active dimeric Drp1-Mff complex, ultimately resulting in mitochondrial fission.

Several lines of evidence support the notion that MIEFs are crucial factors in the recruitment of Drp1 to mitochondria and facilitate the binding of Mff to Drp1. First, both siRNA-based knockdown and CRISPR/Cas9-based knockout revealed that absence of MIEF1/2 diminishes Mff-Drp1 binding and Mff-induced Drp1 accumulation on mitochondria. In contrast, knockdown of Mff did not impair MIEF-Drp1 association and MIEF-mediated recruitment of Drp1 to the MOM, in line with previous reports that MIEFs recruit Drp1 to mitochondria independently of Fis1 and Mff in mouse embryonic fibroblasts (MEFs)^11^ and in a yeast strain lacking all yeast fission factors^10^. Additionally, a previous study reported that engineered lysosome-targeted MIEF1/MiD51 was much more efficient than lysosome-targeted Mff and Fis1 in recruiting Drp1 to the lysosome^16^. Furthermore, by using engineered cytoplasmically localized mutants of MIEFs (MIEF1^Δ1–48^ and MIEF2^Δ1–49^) and Mff (Mff∆C), in which the respective TM domain anchoring in the mitochondrial membrane had been removed, it was revealed that MIEFs but not Mff can interact with Drp1 when in the cytoplasm. Collectively, these findings support the notion that MIEFs play a critical role in controlling the recruitment of Drp1 from the cytoplasm to mitochondria.

Following the recruitment of Drp1 by MIEFs to the MOM, the data presented here further suggest that MIEFs act as adaptors linking Drp1 and Mff together in a trimeric Drp1-MIEF-Mff complex and that endogenous MIEFs can enhance a direct binding of Drp1 to Mff, most likely via potential reassembly of Drp1 from the trimeric Drp1-MIEF-Mff complex to a dimeric Drp1-Mff complex. Although the details of such a reassembly step could not be elucidated, this notion is supported by several observations. Co-IP showed that MIEFs interact independently with both Drp1 and Mff. Sequential co-IP further revealed the existence of a trimeric complex containing Drp1, MIEF and Mff. The use of Drp1 binding-deficient MIEFs (MIEF1^Δ160–169^ and MIEF2^Δ151–160^) and Mff (Mff∆50) allowed us to conclude that MIEFs but not Mff are the interaction partners of Drp1 in such a trimeric complex. By comparing the levels of the dimeric Mff-Drp1 complex in wild-type and *MIEF1/2* ^DKO^ cells (i.e. in the presence and absence of a trimeric Drp1-MIEF-Mff complex), it was revealed that a significant amount of the dimeric Drp1-Mff complex could be formed in wild-type cells, whereas *MIEF1/2* ^DKO^ cells (i.e. in the absence of a trimeric Drp1-MIEF-Mff complex) had a severely decreased level of the dimeric complex (see Fig. 6G). This suggests that the presence of endogenous MIEFs can promote a direct physical binding of Drp1 to Mff, in addition to their function as molecular bridge between Mff and Drp1. In line with this, in the presence of endogenous MIEFs, elevated levels of Mff induced a significant accumulation of Drp1 on mitochondria, whereas absence of endogenous MIEFs greatly reduced Mff overexpression-induced accumulation of Drp1 on mitochondria. Taken together, the data support the view that endogenous MIEFs present Drp1 to Mff, potentially via an adaptor function followed by reassembly of Drp1 from a trimeric Drp1-MIEF-Mff complex to a dimeric Drp1-Mff complex. Although the underlying mechanisms by which endogenous MIEFs facilitate the direct binding of Drp1 to Mff are still poorly understood, several recent studies imply that the molecular state including oligomerization^15, 21^ as well as the GTPase activity of Drp1^17^ are likely to be involved in controlling the selective binding of Drp1 to either MIEFs or Mff, thereby balancing between a MIEF-bound and an Mff-bound Drp1 state.

The proposed model also provides a plausible explanation for why both overexpression and deficiency of MIEFs can cause a mitochondrial fusion phenotype^11–13, 18, 19^. In situations of overexpression, MIEFs recruit Drp1 to mitochondria but high levels of MIEFs sequester Drp1 in the Drp1-MIEF or Drp1-MIEF-Mff complexes, and prevent a physical binding of Drp1 to Mff, thereby inhibiting fission and resulting in a severe mitochondrial fusion phenotype (Fig. 8, ii). In contrast, MIEFs at low-to-moderate levels (e.g. in most endogenous situations) are able to bring Drp1 to the MOM and efficiently transfer it to Mff promoting mitochondrial fission (Fig. 8, iii). However, in the absence of MIEFs, Mff itself cannot capture sufficient amounts of Drp1 to maintain the balance of fission and fusion. As a consequence, mitochondrial dynamics is shifted towards fusion, thereby leading to a moderate mitochondrial elongation phenotype (Fig. 8, iv). Consistent with the model, the data obtained from restoring 293T cells depleted of endogenous MIEFs demonstrate that at low levels, exogenous MIEFs promote mitochondrial fission, but at high levels, the direct binding of Drp1 to Mff is impaired, thereby resulting in a mitochondrial fusion phenotype. In keeping with this notion, in MiD49^−/−^ (MIEF2^−/−^) HCT116 cells, low levels of exogenous Myc-MiD49 can induce mitochondrial fission, while high levels of Myc-MiD49 inhibit fission^22^. It was also reported that low levels of exogenously expressed MiD51/MIEF1 could promote mitochondrial fission, whereas high MiD51/MIEF1 levels blocked fission in wild-type Cos7 cells^16, 23^. Also, we previously reported that knockdown of MIEF1 alone by siRNA caused mitochondrial fission in HeLa cells^13^, whereas complete knockout of endogenous MIEF1 by CRISPR/Cas9 editing led to mitochondrial elongation in HeLa cells^18^. Taken together, the data imply that modulation of MIEF levels impacts on the balance of mitochondrial fusion and fission.

Finally, it should be stressed that although MIEFs in coordination with Mff play a key role in Drp1-mediated fission, the data also show that Mff can recruit Drp1 to mitochondria independently of MIEFs to regulate fission events as described in a yeast strain lacking all yeast fission factors^10^, and loss of MIEFs only partially reduces Mff-mediated mitochondrial fission. However, the ability of Mff to bind Drp1 is markedly impaired, thereby reducing Mff-mediated Drp1 accumulation on mitochondria in the absence of MIEFs. Consistent with this, Palmer *et al*.^16^ showed that engineered lysosome-targeted Mff only led to a low recruitment of Drp1 to the organelle. Conversely, Drp1 was efficiently recruited to the organelle in the presence of lysosome-targeted MIEF1/MiD51^16^. Taken together, this argues that MIEFs play an important role in the recruitment of Drp1 to mitochondria and in the regulation of Mff-Drp1 complex formation on mitochondria. In general, Mff can bind Drp1 independently of MIEFs but MIEFs can enhance the ability of Mff to bind Drp1. Additionally, MIEFs are also likely to act in and regulate other phases of the fission process. For example, it has been reported that MIEFs differentially promote Drp1 oligomerization and GTP hydrolysis, and this function of MIEF1 is enhanced by the cofactor ADP as revealed by crystal structure analysis^24, 25^. It was also reported that MIEF overexpression promotes mitochondrial fission in Mff/Fis1 knockout cells treated with carbonyl cyanide m-chlorophenylhydrazone (CCCP)^11^.

In conclusion, this work sheds new light on the delicate control of mitochondrial dynamics, and we propose that MIEFs and Mff can work both coordinately and independently in regulating the mitochondrial recruitment of Drp1 and Drp1-mediated mitochondrial fission. Further, there is a balance between MIEFs and Mff in their interaction with Drp1 and relative levels of MIEFs and Mff likely provide a potential mechanism to control the balance between mitochondrial fusion and fission in cells. It will further be important to explore to what extent MIEFs and Mff cooperate with other regulatory proteins and cellular structures, such as the endoplasmic reticulum (ER) and actin filaments, which have been implicated in the regulation of mitochondrial fission^26–28^.

## Methods

Cell cultures, transfections, antibodies, other reagents, expression constructs (Table S1), siRNA targeting sequences (Table S2), Western blot, co-immunoprecipitation (co-IP) and the establishment of 293T cell lines with stable low-level expression of either MIEF1-V5 or MIEF2-V5 are all described in detail in the Supplemental Experimental Procedures.

### RNA interference by siRNA

siRNAs were introduced by Lipofectamine^TM^ RNAiMax (Invitrogen) according to the manufacturer’s protocol. Twenty-four hours after initial transfection, 293T cells were re-transfected with the same siRNA, incubated for another 48 h and harvested for further investigations. For siRNA treatment followed by overexpression of target proteins, cultured cells were first treated with siRNA as described. After 48 h of siRNA treatment, cells were transfected with expression plasmids, incubated for another 24 h and harvested for further investigation.

### Generation of MIEF1 and MIEF2 single-knockouts and MIEF1/2 double-knockout 293T cell lines

*MIEF1* ^KO^, *MIEF2* ^KO^ and *MIEF1/2* ^DKO^ 293T cells were established using the CRISPR/Cas9 gene-editing system^20^. The vector used encodes a Cas9 nuclease expression cassette and guide RNA cloning cassette (Addgene plasmid #48139). The *MIEF1* guide RNA and *MIEF2* guide RNA (Supplementary information, Figure S2A) were designed via a web site (crispr.mit.edu). 293T cells were transfected with the CRISPR nuclease vector with the gene-target guide RNA for 24 h, and subsequently the transfected cells were selected with 3 μg/ml puromycin for 48 h, and then incubated in puromycin-free medium overnight. Single cells were picked and cultured in 48-well plates for 2 weeks, and each colony was split into 3 wells of 6-well plates for protein extraction, genomic DNA purification and freezing. The putative *MIEFs* KO cell colonies were validated by Western blotting, and by PCR cloning followed by DNA sequencing (Supplementary information, Figure S2B and S2C).

### Immunofluorescence confocal microscopy

Immunofluorescence confocal microscopy was carried out as described^13, 19^. For mitochondrial staining, the MitoTracker Red CMXRos (500 nM, Molecular Probes) was added to cultures 15 min before fixation. Specimens immunostained with different antibodies were examined in the SP5 confocal microscopy system (Leica). Quantitative co-localization analysis of confocal images was performed with the Pearson’s correlation coefficient (*r*) using the Leica integrated program. The Pearson’s correlation coefficient was presented as mean ± SEM by the Student’s t-test software (http://www.physics.csbsju.edu/stats/t-test.html). Measurement of immunofluorescence intensity was performed by the Leica confocal microscopy software.

### Image processing and analysis

The confocal images for surface rendering were deconvolved by the Autoquant X3 software (Media Cybernetics, Inc.) with the theoretical point spread function (PSF). The signal to noise ratios (SNR) and background were determined automatically. 3D surface rendering was performed using Imaris software (Bitplane AG). The threshold was determined in accordance with the maximum intensity projection (MIP). These surface render templates were saved and used as standard for batch processing images.

### Sequential co-immunoprecipitation (co-IP)

For sequential co-IP, 293T cells were co-transfected with MIEF1-V5 and Myc-Mff, and proteins were *in vivo* cross-linked by adding 1% formaldehyde (FA) in PBS buffer before harvest. Cell lysates were first co-IPed with anti-V5 beads, and the co-IPed complexes on the beads were eluted with 300 µl of lysis buffer containing 250 µg V5-peptide (Sigma) at RT for 1 h and centrifuged. Supernatants were transferred to a new tube and kept on ice, and beads were eluted again with another 300 µl of V5-peptide. The total eluted MIEF1-V5-associated complexes (600 µl) were used for a second co-IP with anti-Myc beads overnight at 4 °C.

For immunodepletion of endogenous MIEFs and MIEF-associated proteins as shown in Fig. 6F, cell lysates from wild-type 293T cells were incubated with Dynabeads® protein G conjugated with rabbit MIEF1- and MIEF2-specific antibodies overnight at 4 °C. As controls in this immunodepletion experiment, cell lysates from wild-type and *MIEF1/2* ^DKO^ 293T cells were incubated with Dynabeads® protein G conjugated with rabbit normal IgG overnight at 4 °C. The resulting supernatants from immunodepletion of endogenous MIEFs and controls were used for co-IP with goat anti-Mff antibody or goat normal IgG (control) overnight at 4 °C, and the different fractions were subjected to immunoblotting analysis.

### Statistical analysis

The unpaired Student’s t-test (http://www.physics.csbsju.edu/stats/t-test.html) was applied to evaluate differences between experimental groups. Standard error of the mean (SEM) was calculated by a web-based software: (http://www.endmemo.com/math/sd.php). *P*-values less than or equal to 0.05 indicated statistical significance.

## Electronic supplementary material

Supplementary Information
