# Supplementary material for: MIEF1/2 function as adaptors to recruit Drp1 to mitochondria and regulate the association of Drp1 with Mff
Source: Sci Rep. 2017 Apr 13;7:880. doi: 10.1038/s41598-017-00853-x (PMC5429825; doi:10.1038/s41598-017-00853-x)
Supplement: Supplementary file 1 — Supplementary Information [file 41598_2017_853_MOESM1_ESM.pdf]

Supplemental information

MIEF1/2 function as adaptors to recruit Drp1 to mitochondria and regulate the association of Drp1 with Mff

Rong Yu[1,3], Tong Liu[1,3], Shao-Bo Jin[2], Chenfei Ning[1], Urban Lendahl[2*], Monica Nistér[1,4*], and Jian Zhao[1,4*]

[1] Department of Oncology-Pathology, Karolinska Institutet, CCK R8:05, Karolinska University Hospital Solna, SE-171 76 Stockholm, Sweden;

[2] Department of Cell and Molecular Biology, Karolinska Institutet, SE-171 77 Stockholm, Sweden

[3] These authors contributed equally to the work

[4] These authors share senior responsibility

Supplemental Figures

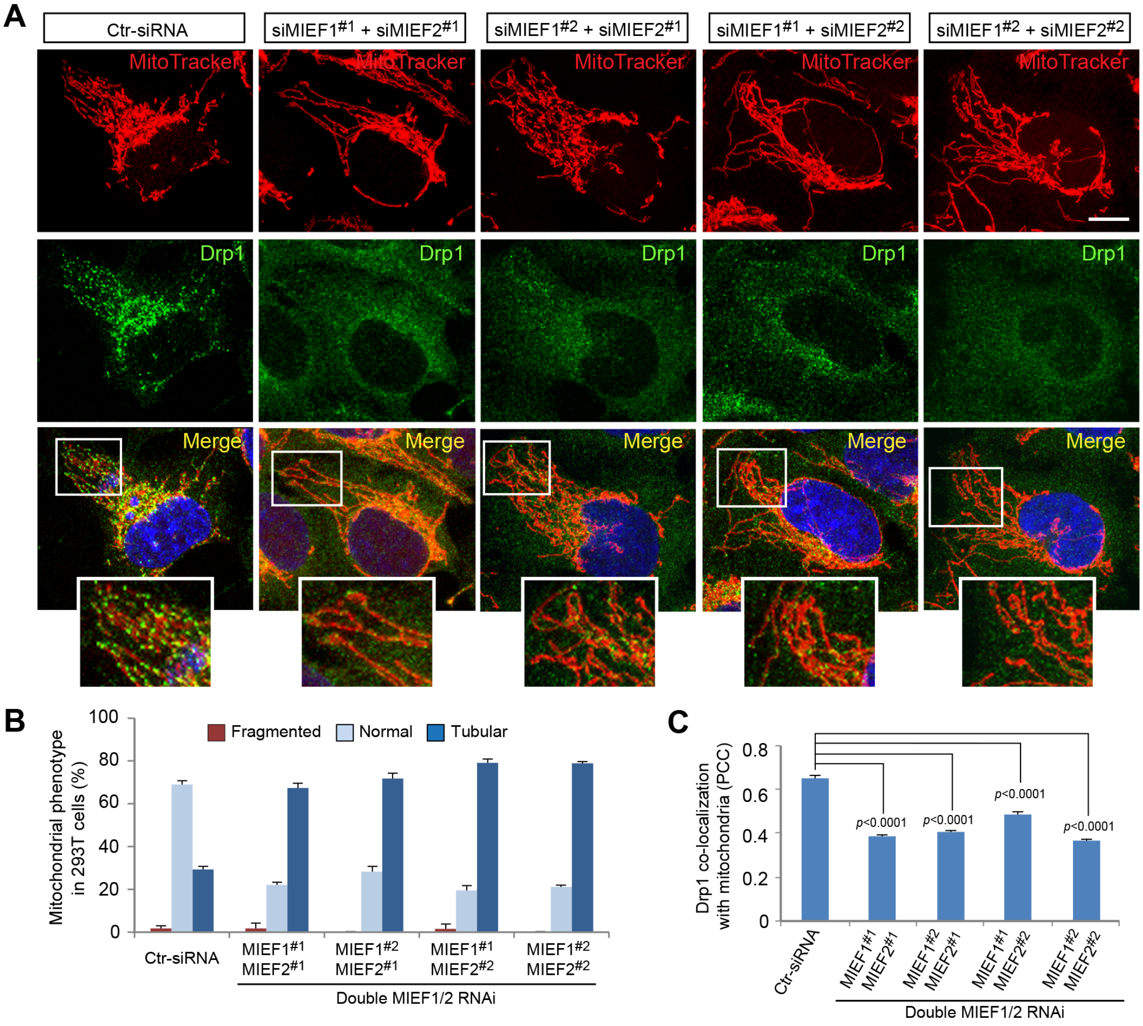

Figure S1. Simultaneous knockdown of MIEF1/2 reduces the amount of Drp1 on mitochondria, leading to mitochondrial elongation.

(A) Confocal images of mitochondrial morphology and Drp1 distribution in 293T cells treated with MIEF1 and MIEF2 siRNAs in different combinations as indicated, and stained with MitoTracker (red) and anti-Drp1 antibody (green). Nuclei were stained with DAPI (blue). Insets represent high magnification views of the boxed areas. Scale bar, 10 µm.

(B) Percentages (mean ± SEM) of cells with indicated mitochondrial morphologies in 293T cells treated with control siRNA, or MIEF1 and MIEF2 siRNAs in combinations as indicated.

(C) Quantitative co-localization of endogenous Drp1 (green) with mitochondria (MitoTracker Red) was analyzed using the Pearson's correlation coefficient (PCC) (mean ± SEM) of at least 5 images (each containing 5-10 cells). The analysis is based on two independent experiments.

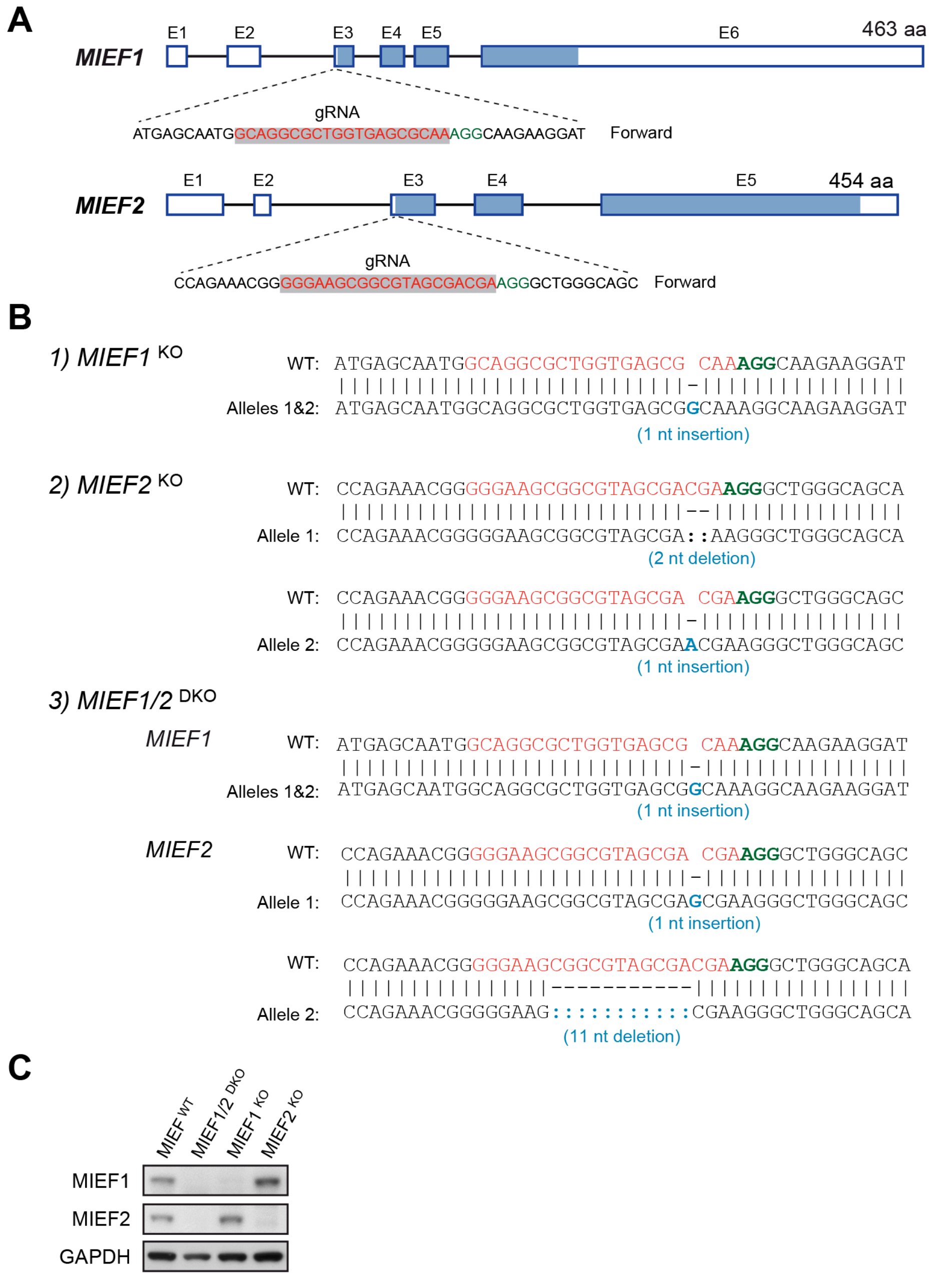

Figure S2. Generation of MIEF1 and MIEF2 single knockout (*MIEF1*^{KO} and *MIEF2*^{KO}) and MIEF1/2 double knockout (*MIEF1/2*^{DKO}) 293T cell lines by CRISPR/Cas9 gene editing

(A) The sequences and locations of single guide RNAs (gRNA, red color) targeted to the human MIEF1 or MIEF2 gene and the corresponding protospacer adjacent motif (PAM, green color) required for CRISPR/Cas9 gene targeting are shown.

(B) Sequence alignments around the edited gRNA targeting sites of wild-type (WT) and mismatches (blue color) in MIEF1- and MIEF2-single knockout and MIEF1/2-double knockout clonal cell lines. The data were obtained from PCR cloning followed by sequencing.

(C) The single cell colonies derived and expanded from *MIEF1*^{KO}, *MIEF2*^{KO} and *MIEF1/2*^{DKO} 293T cells were confirmed by Western blot analysis as indicated.

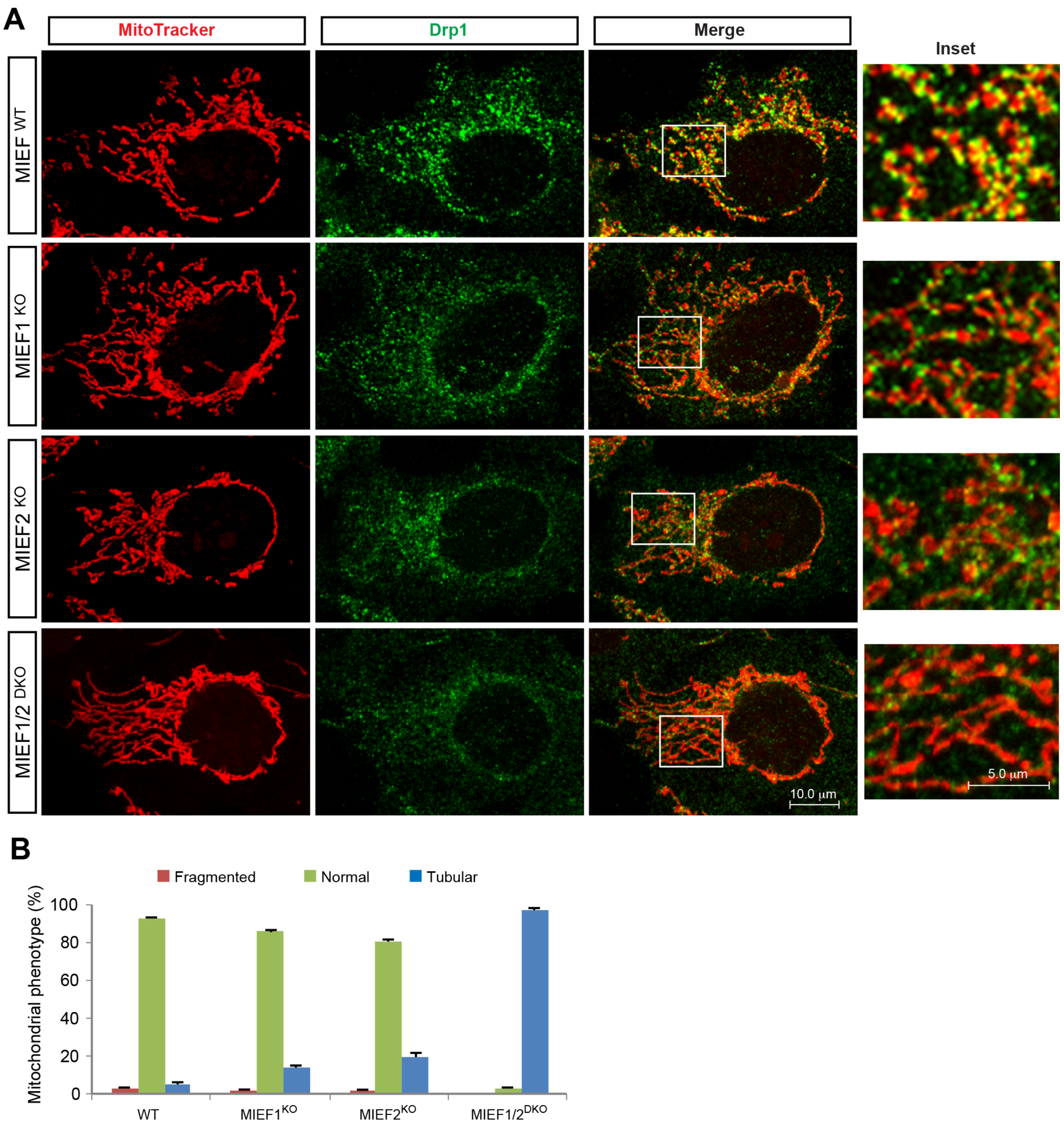

Figure S3. **Knockout of both MIEF1/2 severely decreases Drp1 levels on mitochondria and results in mitochondrial elongation**

(A) Confocal images of mitochondrial morphology and Drp1 distribution in wild-type, *MIEF1*^{KO}, *MIEF2*^{KO} and *MIEF1/2*^{DKO} 293T cells, stained with MitoTracker (red) followed by immunostaining with anti-Drp1 (green) antibody. Insets represent high magnification views of the boxed areas. Quantitative co-localization of Drp1 with mitochondria was analyzed and results are summarized in Figure 3D.

(B) Percentages (mean ± SEM) of cells with indicated mitochondrial morphology in wild-type, *MIEF1*^{KO}, *MIEF2*^{KO} and *MIEF1/2*^{DKO} 293T cell cultures. Data were analyzed from three independent experiments.

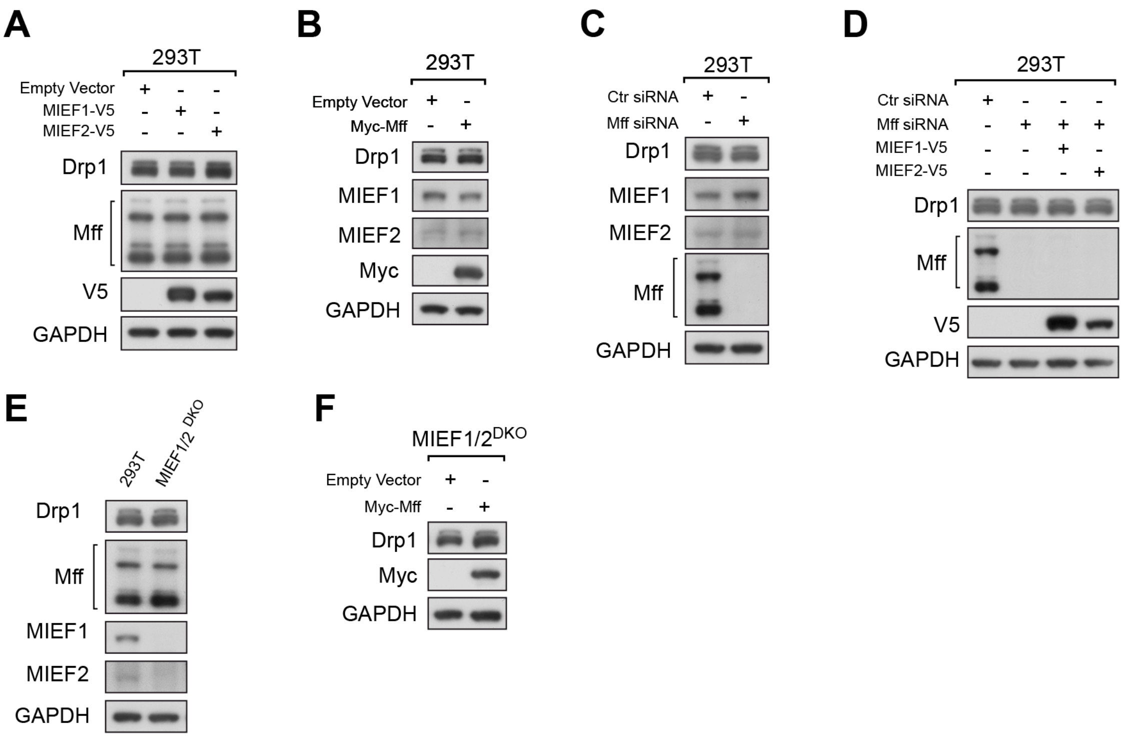

Figure S4. **Levels of Drp1, Mff, MIEF1 and MIEF2 in genetically modified 293T cells as indicated are assessed by Western blotting, and this did not indicate any compensatory effects caused by the modifications**

(A, B) Western blot analysis of 293T cells exogenously expressing MIEF1-V5, MIEF2-V5 (A) or Myc-Mff in (B) respectively, compared to empty vector controls.

(C, D) Western blot analysis of 293T cells treated with Mff siRNA (C) or with Mff siRNA followed by overexpression of either MIEF1-V5 or MIEF2-V5 (D).

(E) Western blot analysis of MIEF1/2^{DKO} cells compared to wild-type control.

(F) Western blot analysis of MIEF1/2^{DKO} cells exogenously expressing Myc-Mff, compared to empty vector control.

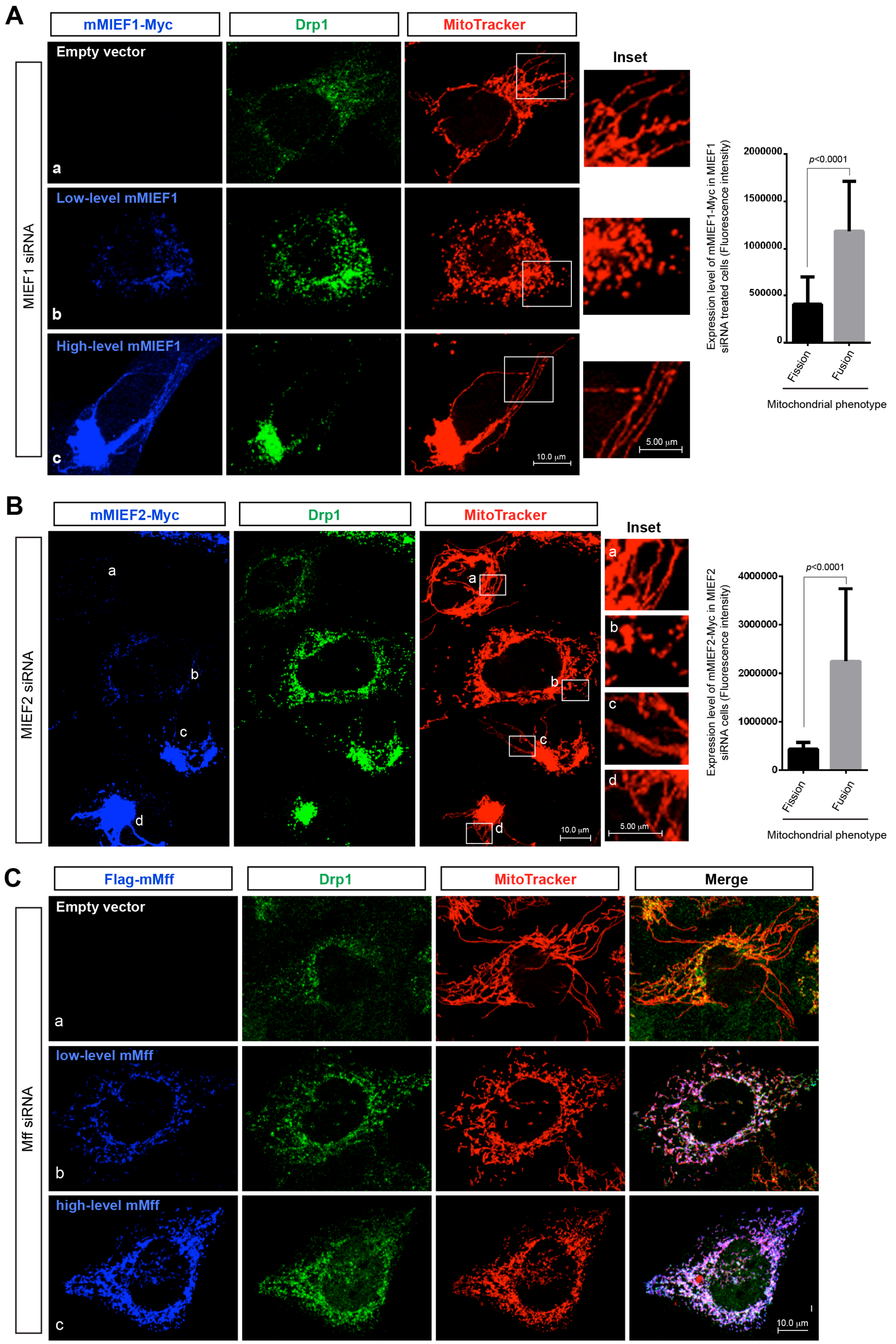

A
mMIEF1-Myc
Drp1
MitoTracker
Inset
MIEF1 siRNA
Empty vector
a
Low-level mMIEF1
b
High-level mMIEF1
c
10.0 µm
5.00 µm
Expression level of mMIEF1-Myc in MIEF1 siRNA treated cells (Fluorescence intensity)
2000000
1500000
1000000
500000
0
p<0.0001
Fission
Fusion
Mitochondrial phenotype
B
mMIEF2-Myc
Drp1
MitoTracker
Inset
MIEF2 siRNA
a
b
c
d
10.0 µm
5.00 µm
Expression level of mMIEF2-Myc in MIEF2 siRNA cells (Fluorescence intensity)
4000000
3000000
2000000
1000000
0
p<0.0001
Fission
Fusion
Mitochondrial phenotype
C
Flag-mMff
Drp1
MitoTracker
Merge
Mff siRNA
Empty vector
a
low-level mMff
b
high-level mMff
c
10.0 µm

Figure S5. Distinct effects of introduced mouse MIEFs or mouse Mff respectively on mitochondrial morphology and Drp1 distribution in cells depleted of endogenous MIEFs or Mff

(A, B) Lower cellular levels of exogenous mMIEF1 and mMIEF2 induced a mitochondrial fission phenotype in cells depleted of endogenous MIEF1 or MIEF2 respectively by siRNA, whereas higher levels of exogenous mMIEFs leads to a mitochondrial fusion phenotype. Confocal images (left panels) of 293T cells treated with either MIEF1 or MIEF2 siRNA, followed by introduction of either mouse MIEF1-Myc (mMIEF1) or mouse MIEF2-Myc (mMIEF2), and stained with MitoTracker (red), anti-Drp1 (green) and anti-Myc (blue) antibodies. Cells marked in **(A)** by (a) to (c) represent: empty vector-transfected cell (a); low-level of mMIEF1-Myc (b); high-level of mMIEF1-Myc (c). Cells marked in **(B)** by (a) to (d) represent: empty vector-transfected cell (a); low-level of introduced mMIEF2-Myc (b); high-level of introduced mMIEF2-Myc (c and d). Insets represent high magnification views of the boxed areas. Expression levels of mMIEF1- and mMIEF2-Myc in transfected 293T cells exhibiting a mitochondrial fission or a fusion phenotype were measured by immunofluorescence intensity (mean ± SD) (right panels) using the ImageJ in at least 10 cells for each condition.

(C) Introduction of exogenous mouse Mff promotes mitochondrial fission irrespective of low or high levels of transfected mMff in cells depleted of endogenous Mff by siRNA. Confocal images of 293T cells treated with Mff siRNA, followed by introduction of mouse Flag-Mff (mMff), and stained with MitoTracker (red), anti-Drp1 (green) and anti-Flag (blue) antibodies. Cells marked by (a) to (c) represent: non-transfected cell (a); low-level of introduced Flag-mMff (b); high-level of introduced Flag-mMff (c).

Supplemental Experimental Procedures

Cell cultures and transfection

The 293T cells were cultured in Dulbecco's modified Eagle's medium (HyClone) with 10% fetal bovine serum. Transient transfection of plasmids was performed using Lipofectamine™ 2000 transfection reagent (Invitrogen) according to the manufacturer´s protocol.

Establishment of 293T cell lines with stable expression of MIEF2-V5

The 293T cells were transfected with the MIEF2-V5 plasmid (pcDNA3.1V5/His) with neomycin (G418) resistance. From the following day, the transfected cells were cultured for two weeks in regular growth medium containing G418 (2 mg/ml) for selection of stably transfected colonies. Cell lines with stable expression of MIEF2-V5 were validated by immunoblotting and immunofluorescence microscopy.

Antibodies and reagents

Mouse monoclonal antibodies (mAbs) used in this study were: V5-tag (Invitrogen); Drp1(DLP1) (BD Biosciences); GAPDH (Santa Cruz); Flag-tag and Myc-tag (Sigma). Rabbit polyclonal antibodies (pAbs) used in this study were: V5-tag (Abcam); Myc-tag and GFP-tag (Sigma); MIEF2 and Mff (Atlas Antibodies); MIEF1 (Zhao et al., 2011) and MIEF2 (Liu et al., 2013); goat polyclonal Mff antibody (T-14), normal goat IgG (Santa Cruz); goat V5-tag polyclonal antibody (Novus Biologicals). Secondary antibodies included the DyLight 488- and 649-conjugated anti-mouse and anti-rabbit IgG antibodies (Vector Laboratories) for immunofluorescence and the peroxidase-conjugated anti-mouse and anti-rabbit IgG antibodies (GE Healthcare). Dynabeads® Protein G for immunoprecipitation, the protein G horseradish peroxidase conjugated antibody (Life technologies) and V5 peptide (Sigma) were used for co-IP experiments.

Expression Constructs

All expression plasmids used in this study, except the GFP-MffΔ50 construct, have been described in previous studies and are listed here in Supplemental Table S1. Empty vectors of pcDNA3.1V5/His and pAcGFP-C In-Fusion® Ready Vector (Clontech) were used as controls.

For generation of a GFP tagged MffΔ50 expression vector lacking the first 1-50 amino acids of Mff, the cDNA was amplified by PCR from the Myc-Mff (isoform 8) expression plasmid (Gandre-Babbe and van der Bliek, 2008) as template using a pair of primers: GFP-Mff fusion-F (5′-AAGGCCTCTGTCGACATGCAAGTTCCGG AGAGGATTG-3′) and GFP-Mff fusion-R (5′-AGAATTCGCAAGCTTCTAGCGGCG AAACCAGAGCCAGCT-3′), and cloned into the pAcGFP1-C In-Fusion® Ready Vector (Clontech) to produce a GFP-tagged MFFΔ50 construct (GFP-MffΔ50). The plasmid was verified by sequencing.

Table S1. Expression constructs used in this study:

Expression construct	Species	Vector	Subcellular localization of expressed protein	Drp1 binding	References
MIEF1-V5	Human	pcDNA3.1V5/His	Mitochondria	Yes	(Zhao et al., 2011)
MIEF1$^{\Delta 1\text{-}48}$-V5	Human	pcDNA3.1V5/His	Cytoplasm	Yes	(Zhao et al., 2011)
MIEF1$^{\Delta 160\text{-}169}$-V5	Human	pcDNA3.1V5/His	Mitochondria	No	(Zhao et al., 2011)
MIEF2-V5	Human	pcDNA3.1V5/His	Mitochondria	Yes	(Liu et al., 2013)
MIEF2$^{\Delta 1\text{-}49}$-V5	Human	pcDNA3.1V5/His	Cytoplasm	Yes	(Liu et al., 2013)
MIEF2$^{\Delta 151\text{-}160}$-V5	Human	pcDNA3.1V5/His	Mitochondria	No	(Liu et al., 2013)
Myc-Mff (isoform 8)	Human	5XMyc tagged Mff in pCS2MT	Mitochondria	Yes	(Gandre-Babbe and van der Bliek, 2008)
GFP-MffΔ50 (isoform 8)	Human	pAcGFP-C in fusion	Mitochondria	No	This study
FLAG-Mff (isoform 8)	Mouse	pcDNA3.1	Mitochondria	Yes	(Otera et al., 2010)
FLAG-MffΔC (isoform 8)	Mouse	pcDNA3.1	Cytoplasm	No	(Otera et al., 2010)
MiD51-Myc (mMIEF1-Myc)	Mouse	pcDNA3.1	Mitochondria	Yes	(Loson et al., 2013)
MiD49-Myc (mMIEF2-Myc)	Mouse	pcDNA3.1	Mitochondria	Yes	(Loson et al., 2013)

RNA interference (RNAi) for gene silencing

Information on specific siRNAs used in this study is listed in the Supplemental Table S2 and as control we used a scrambled Stealth RNAi™ siRNA Negative Control Kit (12935-100) with similar GC content recommended by Invitrogen.

Table S2. The siRNAs used in this study

Human genes	Sequences (5′->3′)	Companies
MIEF1-#1	5′-GCCAAGCAAGCUGCUGUGGACAUAU-3′	Life Technologies
MIEF1-#2	5′-GGAGCAGAACCUGUGGUCAUGUAUU-3′	Life Technologies
MIEF2-#1	5′-UCGAUGCUGACGACCGCCUCCUCUU-3′	Life Technologies
MIEF2-#2	5′-CCCUGGCCGUGAAGCGGUUCAUUGA-3′	Life Technologies
Drp1	5′-CCUGCUUUAUUUGUGCCUGAGGUUU-3′	Life Technologies
Mff	5′ -GGAGUUCCAAAUGCUAGUGUGAUAA-3′	Life Technologies

Western blotting

Protein extracts were separated by electrophoresis using NuPAGE® Novex Bis-Tris Gel (Invitrogen) and transferred to PVDF membranes with Transfer Pack (Bio-Rad). After blocking with 10% nonfat dry milk in PBS, membranes were incubated with primary antibodies followed by the peroxidase-conjugated secondary antibody (GE Healthcare), and immunocomplexes were detected with the Pierce ECL Western Blotting Substrate (Thermo Scientific). Intensity of the bands on Western blots was measured using ImageJ.

Co-immunoprecipitation (co-IP)

Co-IP experiments were carried out as described (Hajek et al., 2007; Liu et al., 2013; Zhao et al., 2011). Briefly, cultured 293T cells were washed with PBS buffer, proteins were *in vivo* cross-linked by incubating the cells in PBS buffer containing 1% formaldehyde (FA) and the cells were scraped off with a rubber scraper and suspended in lysis buffer (PBS containing 1% NP-40 and protease inhibitor cocktail complete EDTA-free) (Roche Diagnostics). The cell suspensions were sonicated and centrifuged to remove insoluble debris. For the co-IP of exogenous proteins, the resulting supernatants were incubated with anti-V5 agarose, anti-Myc agarose, anti-Flag agarose (Novus Biologicals) or anti-GFP mAb-Agarose beads (MBL); For the co-IP of endogenous proteins, 2 μg of antibody against targeting protein was

incubated with Dynabeads® protein G for 1 h. After washing with lysis buffer twice, the antibody-conjugated beads were added to the resulting supernatants. Thereafter the beads were washed with PBS containing 1% NP-40 followed by PBS. The immunocomplexes captured on the agarose beads conjugated with antibody were dissolved in SDS-sample buffer and subjected to immunoblotting.

Supplemental References

Gandre-Babbe, S., and van der Bliek, A.M. (2008). The novel tail-anchored membrane protein Mff controls mitochondrial and peroxisomal fission in mammalian cells. Molecular biology of the cell *19*, 2402-2412.

Hajek, P., Chomyn, A., and Attardi, G. (2007). Identification of a novel mitochondrial complex containing mitofusin 2 and stomatin-like protein 2. The Journal of biological chemistry *282*, 5670-5681.

Liu, T., Yu, R., Jin, S.B., Han, L., Lendahl, U., Zhao, J., and Nister, M. (2013). The mitochondrial elongation factors MIEF1 and MIEF2 exert partially distinct functions in mitochondrial dynamics. Experimental cell research *319*, 2893-2904.

Loson, O.C., Song, Z., Chen, H., and Chan, D.C. (2013). Fis1, Mff, MiD49, and MiD51 mediate Drp1 recruitment in mitochondrial fission. Molecular biology of the cell *24*, 659-667.

Otera, H., Wang, C., Cleland, M.M., Setoguchi, K., Yokota, S., Youle, R.J., and Mihara, K. (2010). Mff is an essential factor for mitochondrial recruitment of Drp1 during mitochondrial fission in mammalian cells. J Cell Biol *191*, 1141-1158.

Zhao, J., Liu, T., Jin, S., Wang, X., Qu, M., Uhlen, P., Tomilin, N., Shupliakov, O., Lendahl, U., and Nister, M. (2011). Human MIEF1 recruits Drp1 to mitochondrial outer membranes and promotes mitochondrial fusion rather than fission. The EMBO journal *30*, 2762-2778.
